# Platelet transfusion in neonatal intensive care units of 22 European countries: a prospective observational study

**DOI:** 10.1016/j.lanepe.2024.101086

**Published:** 2024-10-10

**Authors:** Nina A.M. Houben, Enrico Lopriore, Karin Fijnvandraat, Camila Caram-Deelder, Marta Aguar Carrascosa, Alain Beuchée, Kristin Brække, Francesco Cardona, Anne Debeer, Sara Domingues, Stefano Ghirardello, Ruza Grizelj, Emina Hadžimuratović, Christian Heiring, Jana Lozar Krivec, Jan Malý, Katarina Matasova, Carmel Maria Moore, Tobias Muehlbacher, Miklos Szabó, Tomasz Szczapa, Gabriela Zaharie, Justine de Jager, Nora Johanna Reibel-Georgi, Helen V. New, Simon J. Stanworth, Emöke Deschmann, Charles C. Roehr, Christof Dame, Saskia le Cessie, Johanna van der Bom, Suzanne Fustolo-Gunnink, INSPIRE Study Group, Miguel Alsina-Casanova, Ola Andersson, Rosa Patricia Arias-Llorente, Adeline Berenger, Edyta Bielska, Marioara Boia, André Birkenmaier, Jakub Biros, Anne Laure Blanquart, Tiziana Boggini, Pascal Boileau, Renata Bokiniec, Ilia Bresesti, Katherine Broad, Giacomo Cavallaro, Jennifer Chauvel, Borbála Cseszneki, Carlo Dani, Klaudia Demová, Diana Dornis, Marie-Pierre Duban, Karolina Dziadkowiec-Motyl, Nika Erzen, Eszter Fanczal, Sara Fernández-Castiñeira, Libusa Galuschka, Ellen Gandaputra, Fermín García-Muñoz Rodrigo, Corinna Gebauer, Hélène Grimault, Kristina Grund, Melanie Gsöllpointner, Silvia Gualdi, Brunetta Guaragni, Markus Hahn, Nadja Haiden, Monica Hasmasanu, Daniela Iacob, Mihaela Ivanici, Raphaela Jernej, Tomáš Juren, Karolina Karcz, Lilijana Kornhauser, Barbara Królak-Olejnik, Lena Legnevall, Verena Lehnerer, Emmanuelle Levine, David Ley, María Del Carmen López Castillo, Mariella Magarotto, Silvia Martini, Iwona Maruniak-Chudek, Rita Moita, Anjola Mosuro, Agnieszka Nowicka, Daniel O'Reilly, Manuela Pantea, Alejandro Pérez-Muñuzuri, Tina Perme, Laura Picciau, Simone Pratesi, Sandra Prins, Maurizio Radicioni, Genny Raffaeli, Reyes Roldan-López, Jean-Michel Roué, Beata Rzepecka Węglarz, Greta Sibrecht, Pauline Snijder, Mirta Starčević, Emese Szántó, Liliana Teixeira, Laura Torrejon, Lourdes Urquía Martí, Laurien Vanbuggenhout, Lorenzo Zanetto

**Affiliations:** a Sanquin Research, Sanquin Blood Supply Foundation, Amsterdam, the Netherlands; b Leiden University Medical Centre, Leiden, the Netherlands; c Amsterdam UMC, University of Amsterdam, Emma Children’s Hospital, Pediatric Hematology, Meibergdreef 9, Amsterdam, the Netherlands; d Sanquin Research, Department of Molecular Cellular Hemostasis, Amsterdam, the Netherlands; e La Fe University Hospital, Valencia, Spain; f CHU de Rennes, Rennes, France; g Oslo University Hospital, Oslo, Norway; h Medical University Vienna, Vienna, Austria; i UZ Leuven, Leuven, Belgium; j Centro Materno-Infantil do Norte - Unidade Local de Saúde de Santo António, Porto, Portugal; k Fondazione IRCCS Policlinico San Matteo, Pavia, Italy; l University Hospital Centre Zagreb, University of Zagreb, School of Medicine, Zagreb, Croatia; m University Medical Centre Sarajevo, Sarajevo, Bosnia and Herzegovina; n Department of Neonatal and Paediatric Intensive Care, Copenhagen University Hospital, Rigshospitalet, Copenhagen, Denmark; o University of Ljubljana, Faculty of Medicine, Ljubljana, Slovenia; p University Medical Centre Ljubljana, Ljubljana, Slovenia; q University Hospital Hradec Králové, Hradec Králové, Czech Republic; r Jessenius Faculty of Medicine, University Hospital Martin, Martin, Slovakia; s University College Dublin, Dublin, Ireland; t National Maternity Hospital, Dublin, Ireland; u University Hospital Zurich, Zurich, Switzerland; v Department of Neonatology Semmelweis University, Budapest, Hungary; w II Department of Neonatology, Poznan University of Medical Sciences, Poznan, Poland; x University of Medicine and Pharmacy Iuliu Hatieganu, Cluj-Napoca, Romania; y Charité - Universitätsmedizin Berlin, Berlin, Germany; z NHS Blood and Transplant, London, United Kingdom; aa University of Oxford, Oxford, United Kingdom; ab Karolinska Institute, Stockholm, Sweden; ac National Perinatal Epidemiology Unit, Oxford Population Health, University of Oxford, Oxford, United Kingdom; ad Faculty of Health Sciences, University of Bristol, Bristol, United Kingdom; ae Women’s and Children’s Division, Southmead Hospital, North Bristol NHS Trust, Bristol, United Kingdom

**Keywords:** Platelet transfusion, Thrombocytopenia, Preterm infants, Neonatology, Neonatal intensive care unit, Europe, Epidemiology

## Abstract

**Background:**

Platelet transfusions are given to preterm infants with severe thrombocytopenia aiming to prevent haemorrhage. The PlaNeT2/MATISSE trial revealed higher rates of mortality and/or major bleeding in preterm infants receiving prophylactic platelet transfusions at a platelet count threshold of 50 × 10^9^/L compared to 25 × 10^9^/L. The extent to which this evidence has been incorporated into clinical practice is unknown, thus we aimed to describe current neonatal platelet transfusion practices in Europe.

**Methods:**

We performed a prospective observational study in 64 neonatal intensive care units across 22 European countries between September 2022 and August 2023. Outcome measures included observed transfusion prevalence rates (per country and overall, pooled using a random effects Poisson model), expected rates based on patient-mix (per country, estimated using logistic regression), cumulative incidence of receiving a transfusion by day 28 (with death and discharge considered as competing events), transfusion indications, volumes and infusion rates, platelet count triggers and increment, and adverse effects.

**Findings:**

We included 1143 preterm infants, of whom 71 (6.2%, [71/1143]) collectively received 217 transfusions. Overall observed prevalence rate was 0.3 platelet transfusion days per 100 admission days. By day 28, 8.3% (95% CI: 5.5–11.1) of infants received a transfusion. Most transfusions were indicated for threshold (74.2%, [161/217]). Pre-transfusion platelet counts were above 25 × 10^9^/L in 33.1% [53/160] of these transfusions. There was significant variability in volume and duration.

**Interpretation:**

The restrictive threshold of 25 × 10^9^/L is being integrated into clinical practice. Research is needed to explore existing variation and generate evidence for various aspects including optimal volumes and infusion rates.

**Funding:**

Sanquin, EBA, and 10.13039/501100008873ESPR.

**Research in context**

**Evidence before this study**

We searched Pubmed for randomized trials comparing platelet transfusion thresholds in preterm infants from database inception to May 1st 2024 without language restrictions. The search included the terms “neonate”, “randomized trial”, “platelet transfusion”, “platelet count” and their synonyms. Three trials were identified, with the PlaNeT2/MATISSE trial being the most extensive conducted to date. This trial, published in 2019, reported higher rates of mortality and/or major bleeding in preterm infants receiving prophylactic platelet transfusions at a liberal threshold of 50 × 10^9^/L compared to a restrictive threshold of 25 × 10^9^/L. Despite the need for further research to replicate this finding and unravel the mechanisms underlying the potential harm, these results support restrictive platelet transfusion strategies in preterm infants. We also performed a similar search for international studies describing platelet transfusion practices in preterm infants across Europe, using the terms “neonate”, “Europe”, “platelet transfusion” and their synonyms. Two international surveys were identified, of which the most recent survey conducted in 2020 reported that 47–57% of European NICUs (depending on gestational age) used thresholds higher than 25 × 10^9^/L for preterm infants without clinical bleeding, despite trial evidence.

**Added value of this study**

This is the first prospective study describing platelet transfusion practices across 22 European countries. We found that 8.3% of very preterm infants received at least one platelet transfusion during the first 28 days after birth. The primary indication given for most transfusions was the platelet count threshold. Around one third of transfusions based on platelet count threshold were given above the restrictive PlaNeT2/MATISSE transfusion threshold of 25 × 10^9^/L. We found substantial differences in platelet transfusion volumes, durations, and infusion rates across Europe.

**Implications of all the available evidence**

The restrictive threshold of 25 × 10^9^/L as evaluated in the PlaNeT2/MATISSE trial is increasingly becoming part of managing thrombocytopenia in preterm infants in Europe. Our results highlight ongoing practice variation. Research gaps were identified for areas such as optimal volumes and infusion rates.

## Introduction

Platelet transfusions are administered to preterm infants with severe thrombocytopenia (platelet count below 50 × 10^9^/L) to reduce the risk of major haemorrhage. The PlaNeT2/MATISSE randomized controlled trial on platelet transfusion thresholds, published in 2019, revealed potential harm associated with platelet transfusion.^1^ There were higher rates of mortality and/or major bleeding in preterm infants with a gestational age (GA) below 34 weeks receiving prophylactic platelet transfusions at a liberal threshold of 50 × 10^9^/L compared to a restrictive threshold of 25 × 10^9^/L. Although further research is needed to confirm these findings and to understand the mechanisms underlying the potential harm, this indication of potential harm, combined with the lack of evidence for benefit of liberal transfusion strategies, may justify restrictive platelet transfusion strategies in preterm infants. A European survey conducted in the following year reported that 47–57% of NICUs used thresholds higher than 25 × 10^9^/L for non-bleeding very preterm and extremely preterm infants respectively.^2^ It is not known to what extent the restrictive threshold has since been adopted in cinical practice. Additionally, there is lack of robust evidence on neonatal transfusion thresholds for specific indications such as invasive procedures, and on other aspects of platelet transfusion practice such as optimal transfusion volumes and rates. There is a need for high quality epidemiological data on neonatal transfusion practices to explore variation in practice, to identify research priorities, and to translate into future studies and implementation projects. We therefore conducted a prospective observational study describing the platelet transfusion prevalence and incidence, primary indications for transfusion, platelet count prior to transfusion, volume, and infusion rate of transfusion, platelet count transfusion increment, and transfusion related adverse effects across 64 NICUs in 22 European countries.

## Methods

### Study design and participants

This study was part of the International Neonatal Transfusion Point Prevalence (INSPIRE) study, an international, multicentre, prospective, observational cohort study describing red blood cell (RBC), platelet and plasma transfusion practices in European NICUs.

The Medical Research Ethics Committee of the Leiden University Medical Centre (Leiden, The Netherlands) approved the study protocol (G21.207), followed by national or regional ethics committees in the participating countries. National coordinating neonatologists guided the recruitment process of participating centres in their respective countries. Together we selected a representative sample of NICUs to invite, considering the size of centres, their academic and non-academic status, and surgical versus non-surgical units, aiming to accurately reflect the diversity of centres within their country. The number of centres invited per country was proportional to country population size, aiming to have larger countries contribute more centres than smaller countries, and aiming for at least two participating centres per country. Approximately 77 centres across 22 European countries were invited, all tertiary level NICUs providing continuous life support and comprehensive care for infants born below 32 weeks gestation. Study conduct complied with the Declaration of Helsinki and the General Data Protection Regulation. A parent advisory board, in partnership with the European Foundation for the Care of the Newborn Infant (EFCNI), provided feedback in all stages of the study. The study protocol included all infants with a GA at birth of less than 32 weeks who were admitted to a participating NICU during the study period. Of note, we restricted our study population to infants born before 32 weeks, instead of 34 weeks as applied in the PlaNeT2/MATISSE trial, as very preterm infants <32 weeks were most likely to receive blood transfusions and were at higher risk for severe thrombocytopenia. Parents and guardians provided consent after prior information if required by national or regional legislation. We registered the study protocol (Supplement 1) in the ISRCTN registry (registration number ISRCTN17267090).

### Procedures

We collected data between September 2022 and August 2023 over a six week study period in the respective centres. We included infants at NICU admission, date of consent, or start of study period, whichever occurred last. Study follow-up continued until the first of the following time points: death, discharge, or end of study period. We defined a transfusion event as the administration of a platelet transfusion. For each transfusion event, we collected the primary indication for transfusion, platelet count closest before and after transfusion (within 24 h pre- and post-transfusion), transfusion volume and duration, and possible transfusion associated adverse effects. Furthermore, we recorded occurrence of major bleeding, culture-positive sepsis, necrotizing enterocolitis (NEC), invasive mechanical ventilation, and surgery (definitions in Supplementary Table S1). We collected all study data using a certified electronic Castor database that complies with ICH E6 Good Clinical Practice standards.

### Outcomes

Main study outcome measures included the platelet transfusion day prevalence rate, the patient-mix adjusted transfusion day prevalence rate, and the cumulative incidence of receiving at least one platelet transfusion during the first 28 postnatal days of life adjusted for competing risks of death and discharge. In the cumulative incidence analysis, we only included patients whose follow-up started at their date of birth. We defined a platelet transfusion day as any admission day in the study period on which the patient received at least one platelet transfusion. We defined the platelet transfusion day prevalence rate as the number of platelet transfusion days per 100 NICU admission days. We used this definition to account for the varying lengths of study follow-up between patients which is inherent to the dynamic cohort study design.

Additional study outcomes included primary indications for transfusion, volume, duration, and infusion rate of transfusion, platelet count prior to transfusion, platelet count transfusion increment, and transfusion related adverse effects.

### Statistical analyses

We formulated a statistical analysis plan prior to conducting the analyses (Supplement 2). Patient and centre characteristics are presented as median (interquartile range (IQR)) or frequency (%). To compute the observed prevalence rate, we conducted a meta-analysis to pool centre prevalence rates into subgroup estimates by country, and then into an overall estimate.

For this, we employed a random effects Poisson model using function ‘metarate’ from package ‘meta’ (R statistical software, version 4.1.17, R Core Team, Vienna, Austria, 2021), given that we expected considerable variability between countries.^3^

We calculated patient-mix adjusted prevalence rates per country through a three-step approach. First, we fitted a logistic regression model with transfusion day (yes/no) as dependent variable and eleven clinical covariables as independent variables (sex assigned at birth, GA at birth, birth weight, congenital anomalies, small for gestational age (SGA), major bleeding, necrotizing enterocolitis (NEC), culture-positive sepsis, mechanical ventilation, surgical procedure, postnatal day). This model estimated the probability of receiving at least one platelet transfusion per infant per day. We averaged these predictions to obtain the expected prevalence rate per country. Thereafter, we divided observed transfusion prevalence rates per country by expected prevalence rates per country. Lastly, we multiplied these country observed/expected ratios with the overall observed prevalence rate to obtain patient-mix adjusted prevalence rates.

Additionally, we calculated the cumulative incidence of receiving at least one platelet transfusion during the first 28 days of life, with death and discharge considered as competing events. We only included infants who were followed up from birth in this analysis, using the ‘cuminc’ function from package ‘cmprsk’ (nonparametric Aalen-Johansen estimate, R statistical software).

We computed infusion rate of transfusion (mL/kg/hour) by dividing transfusion volume (mL/kg) by transfusion duration (hours). We compared platelet counts prior to transfusion with the liberal (50 × 10^9^/L) and restrictive (25 × 10^9^/L) threshold as evaluated in the PlaNeT2/MATISSE trial.^1^ We calculated transfusion increment by subtracting platelet count closest after transfusion from platelet count closest before transfusion (all within 24 h pre- and post-transfusion).

We conducted the analysis for the additional study outcome measures using STATA statistical Software (version 16.1, Texas, USA). We made figures using GraphPad Prism (Version 9.3.1, California, USA). We adhered to the STROBE reporting guideline for observational studies.

### Role of the funding source

The funders of the study had no role in study design, data collection, data analysis, data interpretation, or writing of the report.

## Results

### Patients and centres

We included 1143 patients in 64 centres across 22 European countries, with a median study follow-up per patient of 20 days (IQR: 10–35), totaling 24,978 patient days. The median number of patients per centre was 17 (IQR: 11–25). Patients had a median GA at birth of 28 + 2 weeks (IQR: 26 + 2 to 30 + 2 weeks) and a median birth weight of 1030 g (IQR: 780–1350 g). Mortality during study follow-up was 6.3% (72/1143; 6.3%), at a median postnatal age of 8 days (IQR: 4–18). During study follow-up, 37.5% (428/1143) of preterm infants were discharged, at a median postnatal age of 42 days (IQR: 20–68). Re-admission to the NICU after initial discharge during the study period was 1.6% (18/1143). Patient and centre characteristics are provided in Supplementary Tables S3 and S4, respectively.

### Platelet transfusion

**Fig. 1:**
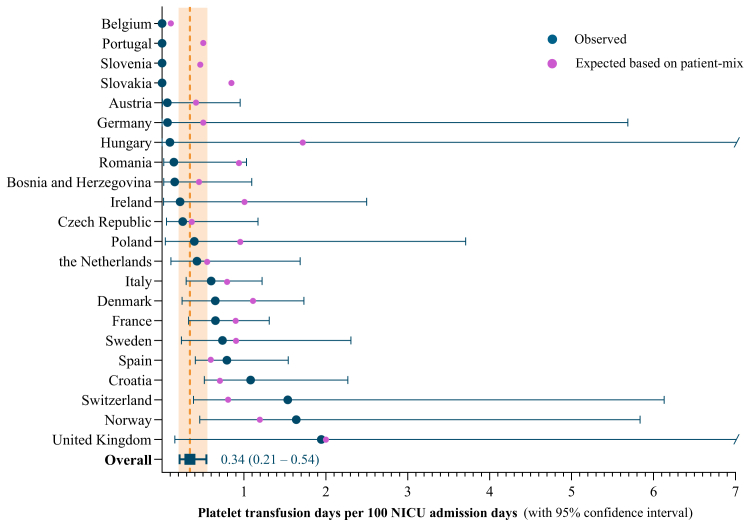
**Overall observed platelet transfusion day prevalence rates, and observed and expected (based on patient mix) prevalence rates per country.** Observed prevalence rates were calculated using random effects Poisson models to pool transfusion day prevalence rates from the individual centres into country subgroup estimates and subsequently to derive the overall estimate. Confidence intervals were in part determined by the sample size per country as well as the practice variation between centres within one country. Upper limit confidence intervals of Hungary and United Kingdom were outside of axis limits. Expected prevalence rates as predicted based on patient-mix using a logistic regression model which included the following variables: sex assigned at birth, GA at birth, birth weight, congenital anomalies, SGA, major bleeding, NEC, sepsis, mechanical ventilation, surgical procedure, postnatal day.

**Fig. 2:**
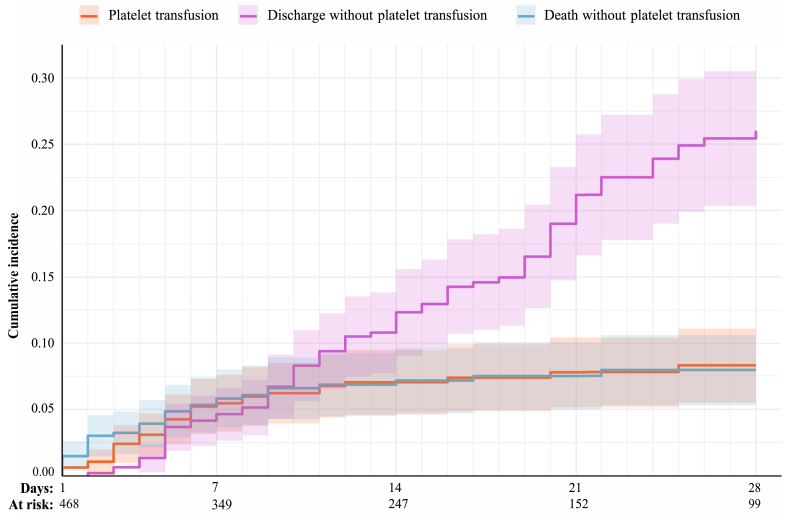
**Cumulative incidence of receiving at least one platelet transfusion during the first 28 postnatal days of life.** Adjusted for the competing risks of death and discharge without platelet transfusion (presented as cumulative incidence, with corresponding 95% confidence intervals). Figure is based on 468 out of 1143 (40.9%) infants that were followed from birth. The number at risk and the number of evenst per postnatal day are available in Supplementary Table S5.

In total, 71 patients (71/1,143, 6.2%) received one or more platelet transfusions, collectively receiving 217 transfusions, with a median of two platelet transfusions per transfused infant (IQR: 1–4). The overall platelet transfusion day prevalence rate was 0.3 platelet transfusion days per 100 admission days (95% CI: 0.2–0.5), corresponding to 3 platelet transfusion days per 1000 admission days. Observed prevalence rates and expected prevalence rates based on patient-mix are presented in Fig. 1. Observed/expected prevalence rate ratios per country ranged from 0.1 to 1.9, indicating that some countries transfuse less (ratio below 1.0) or more (ratio above 1.0) than expected based on case mix (Supplementary Table S4). The proportion of infants that received at least one platelet transfusion by day 7 of life was 5.5% (95% CI: 3.3–7.6), and 8.3% by day 28 of life (95% CI: 5.5–11.1), with death and discharge considered as competing events and based on 468 out of 1143 (40.9%) infants that were followed from birth (Fig. 2).

### Transfusion indication

**Fig. 3:**
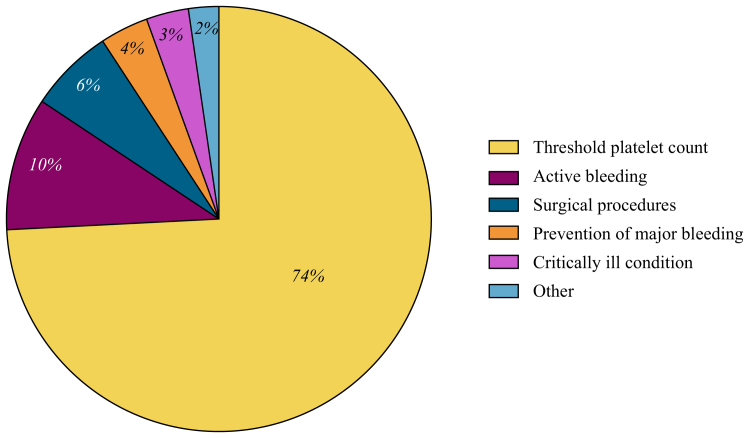
**Primary indications for 217 platelet transfusions.** Indications for transfusion were platelet count threshold (161/217, 74.2%), active bleeding (22/217, 10.1%), surgical procedures (14/217, 6.5%), prevention of major bleeding (based on other factors besides platelet count; 8/217, 3.7%), critically ill conditions (such as NEC and sepsis; 7/217, 3.2%), and other indications (5/217, 2.3%). Other indications included lumbar puncture, urinary catheter placement, low molecular heparin weight treatment for inferior vena cava thrombosis, nonactive bleeding, and petechiae.

The primary indication for most transfusions was platelet count threshold (161/217; 74.2%; Fig. 3). Other primary transfusions indications included active bleeding (22/217, 10.1%), surgical procedures (14/217; 6.5%), prevention of major bleeding (based on other factors besides platelet count; 8/217; 3.7%), critically ill conditions (such as NEC and sepsis; 7/217; 3.2%), lumbar puncture (1/217; 0.5%), urinary catheter placement (1/217; 0.5%), low molecular weight heparin treatment for inferior vena caval thrombosis (1/217; 0.5%), nonactive bleeding (1/217; 0.5%), and petechiae (1/217; 0.5%).

### Transfusion volume, duration and infusion rate

**Fig. 4:**
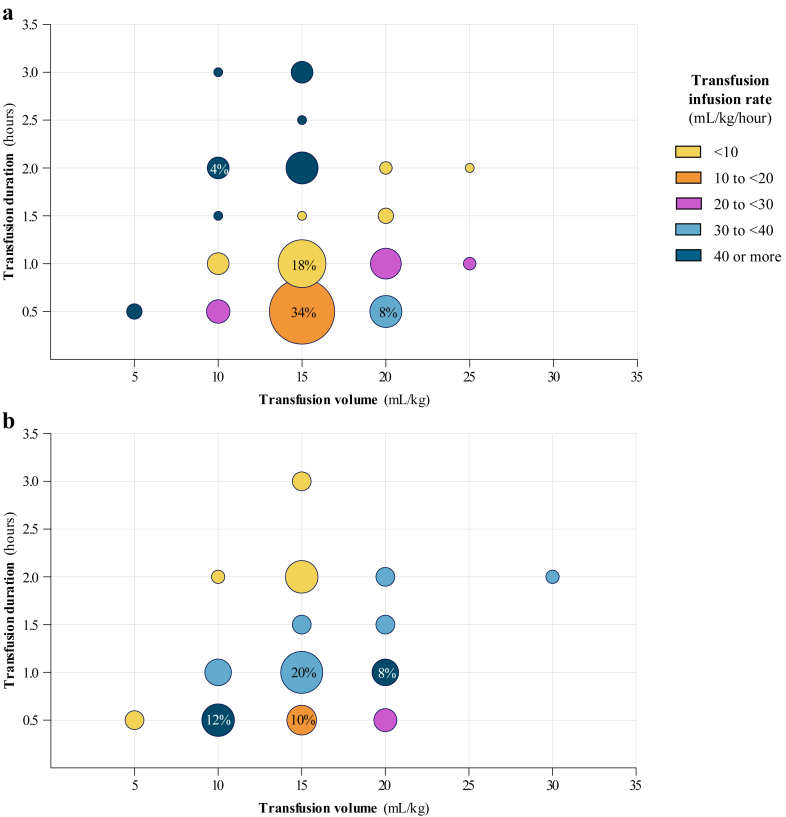
a. Transfusion volume, duration and infusion rate of 161 transfusions given based on threshold. Transfusion volumes were 5 mL/kg or less in 1.9% (3/161), 10 mL/kg in 13.0% (21/161), 15 mL/kg in 64.6% (104/161), 20 mL/kg in 18.6% (30/161), and 25 mL/kg in 1.9% (3/161). Transfusion durations were 30 min or less in 47.8% (77/161), 1 h in 30.4% (49/161), 1.5 h in 3.1% (5/161), 2 h in 13.7% (22/161), 2.5 h in 0.6% (1/161), and 3 h in 4.4% (7/161). Colors reflect most common infusion rate within each dot, infusion rates of individual transfusions within one dot may vary as infusion rates were calculated using unrounded transfusion volumes and durations. Percentages in figure represent the proportion of transfusions indicated for threshold given at this volume and duration. b. Transfusion volume, duration and infusion rate of transfusions given for active bleeding, surgery, prevention of major bleeding, critically ill conditions or any other indication. Volume and duration were available in 50 out of 56 transfusions (89.3%). Colors reflect most common infusion rate within each dot, infusion rates of individual transfusions within one dot may vary as infusion rates were calculated using unrounded transfusion volumes and durations. Percentages in figure represent the proportion of transfusions indicated for active bleeding, surgery, prevention of major bleeding, critically ill conditions or any other indication given at this volume and duration.

For the 161 transfusions given based on threshold, transfusion volumes ranged from 5 to 25 mL/kg, durations ranged from less than 15 min to 3 h, and infusion rates ranged from below 10 to >40 mL/kg/hour (Fig. 4A). The majority of these transfusions had volumes of 15 mL/kg (104/161; 64.6%), followed by 20 mL/kg (30/161; 18.6%). The most common durations were 30 min (77/161; 47.8%) and 1 h (49/161; 30.4%). The most common infusion rates were 10–20 mL/kg/hour (46/161; 28.6%) and 30–40 mL/kg/hour (44/161; 27.3%). Data on transfusions given for active bleeding, surgery, prevention of major bleeding, critically ill conditions or any other indication are provided in Fig. 4B.

### Platelet count prior to transfusion

**Fig. 5:**
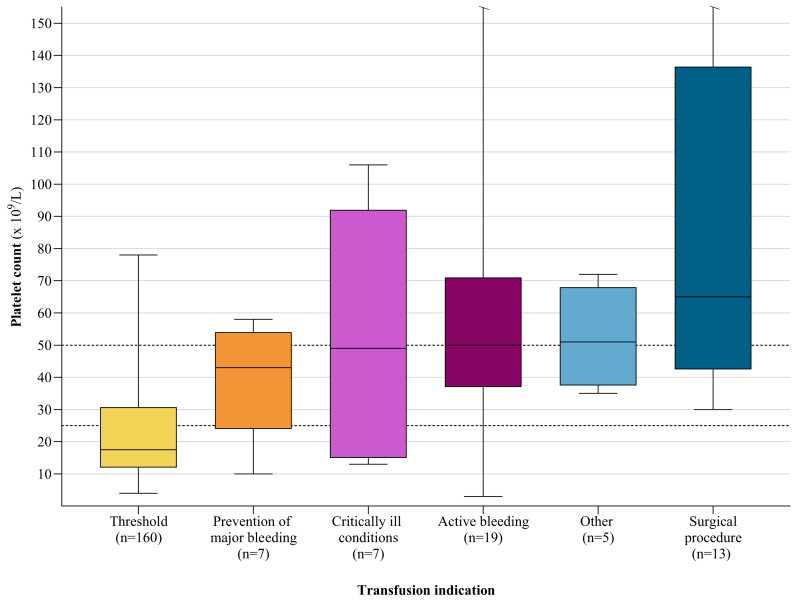
**Platelet count prior to transfusion.** Platelet counts prior to transfusion were available in 211 out of 217 transfusions (97.2%). The dotted lines represent the liberal transfusion threshold of 50 × 10^9^/L and restrictive transfusion threshold of 25 × 10^9^/L as evaluated in the PlaNeT2/MATISSE trial. The middle line of each box denotes the median, the ends of the box denote the 25th to 75th percentile, the whiskers denote the minimum to maximum range. Platelet count prior to transfusion given for active bleeding (n = 1 transfusion) and surgical procedure (n = 3 transfusions) outside axis limits, respectively.

Platelet counts within 24 h pre-transfusion were available for 211 out of 217 (97.2%) transfusions. This included 160 out of 161 (99.3%) transfusions given based on threshold (Fig. 5). The median platelet count prior to transfusion given based on threshold was 18 × 10^9^/L (IQR: 12–31). Compared to the thresholds used for non-bleeding infants in PlaNeT2/MATISSE trial, 107 (107/160; 66.9%) of these transfusion had a platelet count below the restrictive threshold of 25 × 10^9^/L, 45 (45/160; 28.1%) between the restrictive and below the liberal threshold (25–50 × 10^9^/L), and 8 (8/160; 5.0%) were given at or above the liberal threshold of 50 × 10^9^/L. Median platelet counts prior to transfusion given for indications other than threshold ranged from 43 × 10^9^/L to 65 × 10^9^/L. Transfusion indications stratified by pre-transfusion platelet counts below 25 × 10^9^/L or equal to or above 25 × 10^9^/L are available in Supplementary Table S6.

### Transfusion increment

Platelet count increment data were available in 131 out of 161 (81.4%) transfusions given based on threshold. The median increment was 20 × 10^9^/L per transfusion (IQR: 4–52) (Supplementary Fig. S1). We also plotted the association between platelet count increment and transfusion volume (Supplementary Fig. S1).

### Transfusion associated adverse effects

No adverse effects potentially linked to a preceding platelet transfusion were reported during the study period.

## Discussion

To the best of our knowledge, this is the first prospective study describing platelet transfusion practices in preterm infants across Europe. We found varying platelet transfusions rates across the 22 participating countries, with one in three platelet transfusions indicated for platelet counts above the restrictive PlaNeT2/MATISSE threshold of 25 × 10^9^/L and widely varying transfusion volumes and durations.

Several national studies have previously assessed platelet transfusion practices following publication of the PlaNeT2/MATISSE trial, reporting proportions ranging from 5.0 to 11.9% of preterm infants receiving at least one platelet transfusion, similar to the 8.3% we observed.^4^, ^5^, ^6^, ^7^, ^8^

The majority of transfusions (161/217; 74.2%) were indicated primarily based on platelet count threshold. These transfusions are likely to have been given prophylactically with the aim of reducing the risk of bleeding, although their effectiveness in preventing bleeding in preterm infants has never been conclusively established.^9^^,^^10^ Furthermore, there is no straightforward causal link between the degree of thrombocytopenia and bleeding risk, and it is known that bleeding is a multifactorial process influenced by various other factors in addition to platelet count.^9^^,^^11^, ^12^, ^13^, ^14^ This complexity is evident from the fact that not all infants with severe thrombocytopenia bleed, and not all infants with major bleeding have severe thrombocytopenia.^15^

Two thirds of the transfusions primarily based on platelet count were administered when counts were below 25 × 10^9^/L (66.9%), suggesting that the restrictive threshold found beneficial in the PlaNeT2/MATISSE trial is being integrated into European neonatal intensive care. In a 2020 survey, 47–57% of European NICU still reported using thresholds above this restrictive threshold for very preterm as well as extremely preterm infants.^2^ Another retrospective study including both term and preterm infants in the United States, published in 2021, reported a median platelet count of 71 × 10^9^/L prior to prophylactic and therapeutic platelet transfusions combined.^16^ In our study, the median platelet count prior to platelet transfusion indicated by platelet was 18 × 10^9^/L, which would be consistent with a threshold of 25 × 10^9^/L being used in practice. Additionally, some centres may apply even more restrictive thresholds such as 20 × 10^9^/L for non-bleeding infants.^2^ Without further evidence, there remains clinical equipoise in terms of safety and effectiveness of transfusion thresholds below 25 × 10^9^/L for non-bleeding infants.

While our findings suggest progress in the integration of a restrictive prophylactic platelet transfusion threshold in Europe, more than 30% of transfusions were still given for platelet counts above 25 × 10^9^/L, some even exceeding the liberal threshold of 50 × 10^9^/L. This suggests that the restrictive threshold has not been adopted in clinical practice by all centres. The exact reasons for this ongoing threshold variation across practices are not well known. Possibly, dissemination of PlaNeT2/MATISSE trial results may be incomplete or guidelines have not been updated following publication. Moreover, clinicians may have deviated from applied restrictive thresholds due to various reasons. Notably, only 37% of infants in the PlaNeT2/MATISSE trial were enrolled before or on day 5 of life, which may have led some clinicians to be hesitant to adhere to the restrictive threshold during these first days of life.^1^^,^^17^ For this specific reason, a new RCT is currently in preparation in the US to compare restrictive versus liberal transfusion thresholds for extremely preterm infants during the first week of life and beyond (Neonatal Platelet Transfusion Threshold Trial (NeoPlaTT), NIH RePORTER #1UG3HL173303-01). Alternatively, clinicians may have anticipated a subsequent decline in platelet count, prompting an early transfusion decision above the restrictive threshold level. However, given the evidence for harmful short- and long-term effects of higher platelet transfusion thresholds, the use of prophylactic platelet transfusions for platelet counts above 25 × 10^9^/L, in the absence of active bleeding or surgical procedures, should be discouraged.^1^^,^^18^ Of note, we only recorded the primary indication for transfusion, so secondary reasons other than platelet count may also have contributed to the transfusion decision. As platelet count alone may not be a reliable predictor of bleeding, this may have impacted clinical decision-making in various scenarios.

Notably, approximately 10% of transfusions were given therapeutically to preterm infants experiencing active bleeding, aiming to improve primary hemostasis by increasing the platelet count. We currently lack robust evidence concerning optimal transfusion thresholds for active bleeding and other indications such as surgical or invasive procedures. We observed marked variability in platelet counts prior to transfusion given for active bleeding as well as surgical procedures, consistent with this limited evidence base. The design of further studies in these areas of therapeutic practice is challenging, but given the potential risks for harm with platelet transfusions, a better pathophysiological understanding is needed.

We found that in 20.5% (33/161) of platelet transfusions indicated for low platelet count, volumes of 20 mL/kg or higher were administered. In this context, it should be noted that neonatal platelet transfusions are most frequently given prophylactically to euvolemic infants without clinical bleeding. In comparison, adults usually receive around 200–300 mL platelet transfusions, equivalent to approximately 5 mL/kg or less. The most common duration of platelet transfusions for those transfused based on platelet count was 30 min (77/161, 47.8%), resulting in almost half of transfusions given at infusion rates of either 30 or 40 mL/kg/hour. Platelet transfusions in adult patients are also commonly administered over 30 min, probably for historical considerations including minimising time without platelet agitation, although this is a relatively short period compared to accepted platelet transportation non-agitation limits.^19^ However, due to the relatively higher volumes administered to infants in relation to body weight, these short durations result in much higher infusion rates per kg body weight for preterm infants.^20^ The short transfusion durations may be beneficial for patients with clinical bleeding by achieving higher platelet counts more rapidly. However, preterm infants have a fragile cerebral germinal matrix and rapid volume expansion could theoretically harm this by disrupting cerebral or abdominal blood flow, potentially contributing to the development of intraventricular haemorrhage or NEC.^12^^,^^21^ In infants, RBC transfusions are usually given over much longer durations to mitigate potential adverse effects of rapid transfusion administration. It is unclear why similar precautions are not applied with platelet transfusions in preterm infants. Only one small randomized study has compared platelet transfusion durations of 30 min and 2 h in preterm infants in relation to platelet count increments, showing that longer transfusion durations yielded similar platelet transfusion increments.^22^ Large comparative studies are urgently needed to evaluate the efficacy and tolerance of transfusion volumes and infusion rates in non-bleeding infants, and to unify current practices. The use of hyper concentrated platelet transfusions could be beneficial for preterm infants by mitigating these fluctuations caused by high volumes.^23^^,^^24^ A single participating centre in our study used hyper concentrated platelet components.^25^ Further research should clarify whether smaller volumes are safer and effective.

We did not document any immediate adverse effects potentially associated with platelet transfusion. Platelet transfusions are reported to contribute more frequently to transfusion related adverse effects in adult and paediatric patients compared to RBC transfusions.^26^ Preterm infants are inherently susceptible to potential transfusion-related adverse effects given their immaturity. In preterm infants, severe thrombocytopenia is often associated with platelet consumptive and platelet activating diseases such as sepsis or NEC (possibly one of the reasons for the relatively low platelet increments shown in this study).^27^ This complexity may hamper the recognition of platelet transfusion-related adverse effects, in particular if infants receive multiple interventions at the same time. Thus, it seems likely that adverse effects are underrecognized and underreported. Apart from potential short-term harm associated with platelet transfusion, there is also evidence for long-term harm. The PlaNeT2/MATISSE trial reported higher rates of the secondary outcome bronchopulmonary dysplasia (BPD) in the 50 × 10^9^/L transfusion threshold arm as well as short-term harm (major bleeding and/or mortality day 28 of life).^1^ Platelets also play a crucial role in host immunity and release various immune response modifiers, including those that are found only in adult platelets and can contribute to the development of BPD.^28^, ^29^, ^30^, ^31^ There is evidence that platelet transfusions can provoke inflammatory responses or amplify existing inflammation in preterm infants, a potential mechanism leading to long-term harm.^32^, ^33^, ^34^, ^35^, ^36^

Study limitations include the relatively small number of infants included per country, partly due to the six week study period per centre, limiting the interpretation of results at the national level. We did not collect information on the component specifications or platelet counts of the platelet components transfused, known to influence transfusion platelet count increment. Additionally, we did not assess the use of alternative hemostatic therapies such as tranexamic acid and coagulation factor concentrates.

In conclusion, we found the restrictive PlaNeT2/MATISSE transfusion threshold of 25 × 10^9^/L is being integrated into clinical practice. Yet, one third of transfusions based on platelet count threshold as primary indication were given above this threshold of 25 × 10^9^/L. Our results highlight the ongoing practice variation and underline the need for further evidence on various aspects of platelet transfusion practices including optimal volumes and infusion rates.

## Contributors

NH, EL, KF, HVN, SJS, ED, CCR, CD, JB, SFG have designed the study. SFG obtained funding. NH, EL, KF, CCD, MAC, AB, KB, FC, AD, SD, SG, RG, EH, CH, JLK, JM, KM, CMM, TM, MS, TS, GZ, NRG, ED, CCR, CD, SC, JB, SFG contributed to the data collection and/or helped interpret the data. NH and JJ coordinated the study, EL, KF, CCD, JB, SFG supervised the project. NH, CCD, MAC, AB, KB, FC, AD, SD, SG, RG, EH, CH, JLK, JM, KM, CMM, TM, MS, TS, GZ, NRG, ED, CCR, CD provided administrative, technical and/or material support. NH, CCD, SC, JB wrote the statistical analysis plan and performed the statistical analysis. NH and CCD have directly accessed and verified the underlying data reported in the manuscript. NH, EL, KF, SFG wrote the first draft of the manuscript. All authors critically reviewed the draft manuscript and approved the final version prior to submission.

## Data sharing statement

No consent was obtained for further data sharing. External applications for data access will therefore only be taken into consideration after the prior written consent of all participating centres.

## Declaration of interests

CH and TM have received compensation from Sanquin Blood Supply Foundation. JM has received compensation from Sanquin Blood Supply Foundation, research grants from Cooperatio and Personmed, and consulting fees from Danone, Nestlé, Baxter, and Chiesi. All other authors declare no competing interests.
