# Supplementary material for: Platelet transfusion in neonatal intensive care units of 22 European countries: a prospective observational study
Source: Lancet Reg Health Eur. 2024 Oct 10;47:101086. doi: 10.1016/j.lanepe.2024.101086 (PMC11635195; doi:10.1016/j.lanepe.2024.101086)
Supplement: Supplementary Data [file mmc1.pdf]

# Supplementary Data

**Supplementary Table 1.** Definitions

**Supplementary Table 2.** Patient characteristics

**Supplemtary Table 3.** Participating centres characteristics

**Supplementary Table 4.** Patient-mix adjusted platelet transfusion day prevalence rates

**Supplementary Table 5.** Number at risk table for the cumulative incidence of receiving at least one platelet transfusion during the first 28 postnatal days of life

**Supplementary Table 6.** Transfusion indications stratified by pre-transfusion platelet counts below $25x10^9$/L or equal to or above $25x10^9$/L

**Supplementary Figure 1.** Platelet count transfusion increment stratified per transfusion volume

**Supplement 1.** Study protocol

**Supplement 2.** Statistical Analysis Plan

**Supplement 3.** INSPIRE Study Group

| Event | Definitions |
|---|---|
| **Major congenital anomalies** | Chromosomal anomalies, syndromes likely affecting long-term outcome, major malformations requiring surgical correction during newborn period, or cyanotic heart defects |
| **Bleeding disorder** | Any genetic or congenital disorders related to a higher risk of bleeding |
| **Major bleeding** | Any of the following bleedings:<br>– Intraventricular haemorrhage (IVH) is defined as IVH Grade 3 (extension of bleeding involving >50% of ventricular area or dilation of ventricle) or IVH Grade 4/IPE (extension of bleeding into surrounding parenchyma) [1]<br>– Intracranial haemorrhage (non-IVH) is defined as a major bleeding if any of the following apply: neurosurgical intervention is required; radiological imaging showing a midline shift; clinical signs and symptoms of an oxygen deficit with significant derangement of laboratory investigations<br>– Pulmonary bleeding is defined as acute fresh blood through the endotracheal tube associated with increased ventilatory requirements or the need for intubation and ventilation<br>– Frank rectal bleeding is defined as macroscopic faecal bleed (not if only occult positive) |
| **Sepsis** | Culture-positive sepsis |
| **NEC** | At least NEC Stage III according to the Modified Bell Staging Criteria [2] |
| **Invasive mechanical ventilation** | Any form of invasive respiratory support for which the neonate is intubated, including conventional mechanical ventilation and high frequency oscillation (HFO). |
| **Transfusion associated adverse effect** | Any adverse effects that the local investigator deemed potentially associated with the preceding transfusion. |
| **Small for gestational age (SGA)** | Birth weight below $10^{th}$ percentile [3] |

**Supplementary Table 1. Definitions**

| **Country** | **Patients** (n (% of total)) | **Follow-up,** in days (n (% of total)) | **Follow-up per patient,** in days (median (IQR)) | **Postnatal age at inclusion,** in days (median (IQR)) | **Female sex assigned at birth** (n (%)) | **Gestational age at birth,** in weeks + days (median (IQR)) | **Birth weight,** in grams (median (IQR)) | **IUGR** (n (%)) | **Congenital anomalies** (n (%)) | **Major bleeding*** (n (%)) | **NEC*** (n (%)) | **Sepsis*** (n (%)) | **Invasive mechanical ventilation**** (n (%)) | **Surgery*** (n (%)) |
|---|---|---|---|---|---|---|---|---|---|---|---|---|---|---|
| Austria | 50 (4.4) | 1397 (5.6) | 29 (14-42) | 13 (1-36) | 22/50 (44) | 26+4 (24+4-28+5) | 890 (650-1280) | 12/50 (24) | 2/50 (4) | 5/50 (10) | 0/50 (0) | 7/50 (14) | 12/50 (24) | 5/50 (10) |
| Belgium | 16 (1.4) | 283 (1.1) | 13 (6-32) | 8 (43-80) | 9/16 (56) | 29+6 (25+4-30+6) | 1180 (852-1357) | 7/16 (44) | 0/16 (0) | 0/16 (0) | 0/16 (0) | 0/16 (0) | 1/16 (6) | 1/16 (6) |
| Bosnia and Herzegovina | 32 (2.8) | 648 (2.6) | 22 (10-31) | 1 (1-21) | 17/32 (53) | 29+6 (28+2-31+1) | 1252 (1015-1592) | 11/32 (34) | 2/32 (6) | 3/32 (9) | 0/32 (0) | 3/32 (9) | 6/32 (19) | 1/32 (3) |
| Croatia | 24 (2.1) | 647 (2.6) | 32 (18-34) | 1 (1-23) | 17/24 (71) | 30+3 (28+1-31+4) | 1225 (1075-1440) | 9/24 (38) | 0/24 (0) | 1/24 (4) | 0/24 (0) | 2/24 (8) | 3/24 (13) | 1/24 (4) |
| Czech Republic | 53 (4.6) | 1173 (4.7) | 18 (13-32) | 5 (1-30) | 21/53 (40) | 30+1 (27+5-31+2) | 1230 (925-1600) | 20/53 (38) | 2/53 (4) | 1/53 (2) | 0/53 (0) | 3/53 (6) | 10/53 (19) | 3/53 (6) |
| Denmark | 34 (3.0) | 615 (2.5) | 14 (5-24) | 3 (1-44) | 14/34 (41) | 27+0 (25+5-29+0) | 940 (788-1200) | 10/34 (29) | 3/34 (9) | 3/34 (9) | 1/34 (0) | 6/34 (18) | 18/34 (53) | 7/34 (21) |
| France | 116 (10.2) | 2664 (10.7) | 21 (11-37) | 4 (1-32) | 50/116 (43) | 28+4 (26+2-30+2) | 1047 (780-1335) | 48/116 (41) | 3/116 (3) | 10/116 (9) | 4/116 (0) | 19/116 (16) | 40/116 (35) | 9/116 (8) |
| Germany | 76 (6.7) | 1951 (7.8) | 25 (13-42) | 17 (1-62) | 33/76 (43) | 26+4 (25+2-29+4) | 845 (605-1190) | 30/76 (40) | 5/76 (7) | 2/76 (3) | 0/76 (0) | 5/76 (7) | 20/76 (26) | 6/76 (8) |
| Hungary | 39 (3.4) | 757 (3.0) | 18 (13-27) | 3 (1-18) | 18/39 (46) | 27+2 (26+1-30+2) | 920 (750-1160) | 18/39 (46) | 1/39 (3) | 6/39 (15) | 3/39 (8) | 13/39 (33) | 13/39 (33) | 2/39 (5) |
| Ireland | 61 (5.3) | 1178 (4.7) | 16 (6-32) | 1 (1-13) | 29/61 (48) | 26+4 (25+2-29+4) | 1090 (850-1400) | 14/61 (23) | 1/61 (2) | 5/61 (8) | 6/61 (10) | 7/61 (11) | 31/61 (51) | 5/61 (8) |
| Italy | 124 (10.9) | 3178 (12.7) | 27 (12-41) | 21 (1-51) | 46/124 (37) | 28+4 (26+4-30+0) | 1050 (817-1290) | 55/124 (44) | 3/124 (2) | 11/124 (9) | 3/124 (2) | 9/124 (7) | 36/124 (29) | 13/124 (10) |
| Netherlands | 79 (6.9) | 1280 (5.1) | 10 (6-24) | 1 (1-10) | 32/79 (41) | 28+4 (26+3-30+4) | 1145 (886-1500) | 17/79 (22) | 2/79 (2) | 4/79 (5) | 0/79 (0) | 8/79 (10) | 25/79 (32) | 4/79 (5) |
| Norway | 33 (2.9) | 567 (2.3) | 12 (5-32) | 5 (1-31) | 15/33 (45) | 26+0 (25+1-26+6) | 793 (636-1010) | 8/33 (24) | 0/33 (0) | 8/33 (24) | 1/33 (3) | 5/33 (15) | 18/33 (55) | 4/33 (12) |
| Poland | 84 (7.4) | 1969 (7.9) | 27 (9-37) | 20 (1-41) | 33/84 (39) | 28+1 (26+6-30+1) | 1085 (850-1345) | 28/84 (33) | 3/84 (4) | 10/84 (12) | 3/84 (3) | 12/84 (14) | 36/84 (43) | 14/84 (17) |
| Portugal | 19 (1.7) | 364 (1.5) | 19 (5-29) | 15 (3-49) | 12/19 (63) | 29+5 (28+6-31+1) | 990 (810-1410) | 10/19 (53) | 0/19 (0) | 0/19 (0) | 0/19 (0) | 2/19 (10) | 4/19 (21) | 0/19 (0) |
| Romania | 29 (2.5) | 688 (2.8) | 23 (14-33) | 4 (1-12) | 15/29 (52) | 29+0 (27+4-31+0) | 1300 (880-1600) | 8/29 (28) | 3/29 (10) | 7/29 (24) | 1/27 (3) | 4/29 (14) | 17/29 (59) | 0/29 (0) |
| Slovakia | 30 (2.6) | 783 (3.1) | 27 (12-39) | 7 (3-38) | 17/30 (57) | 27+6 (25+6-30+2) | 940 (780-1280) | 13/30 (43) | 2/30 (7) | 0/30 (0) | 1/30 (3) | 3/30 (10) | 9/30 (30) | 1/30 (1) |
| Slovenia | 20 (1.8) | 359 (1.4) | 15 (7-26) | 23 (1-55) | 10/20 (50) | 28+3 (26+4-29+6) | 895 (730-1347) | 12/20 (60) | 1/20 (5) | 1/20 (5) | 0/20 (0) | 0/20 (0) | 6/20 (30 | 1/20 (1) |
| Spain | 87 (7.6) | 1771 (7.1) | 17 (10-31) | 1 (1-23) | 36/87 (41) | 28+5 (26+4-30+2) | 1000 (770-1285) | 44/87 (51) | 1/87 (1) | 5/87 (6) | 2/87 (0) | 9/87 (10) | 16/87 (18) | 6/87 (7) |
| Sweden | 49 (4.3) | 830 (3.3) | 15 (5-26) | 4 (1-11) | 20/49 (41) | 28+3 (25+5-29+6) | 1060 (745-1396) | 17/49 (35) | 4/49 (8) | 3/49 (6) | 5/49 (10) | 2/49 (4) | 22/49 (45) | 6/49 (12) |
| Switzerland | 50 (4.4) | 1010 (4.0) | 17 (8-33) | 2 (1-18) | 24/50 (48) | 28+4 (26+3-30+4) | 1120 (800-1470) | 19/50 (38) | 3/50 (6) | 6/50 (12) | 1/50 (2) | 2/50 (4) | 19/50 (38) | 2/50 (4) |
| United Kingdom | 38 (3.3) | 866 (3.5) | 23 (12-36) | 3 (1-20) | 12/38 (32) | 26+6 (24+3-29+2) | 883 (618-1120) | 15/38 (39) | 2/38 (5) | 1/38 (3) | 2/38 (5) | 4/38 (10) | 21/38 (55) | 7/38 (18) |
| **Overall** | **1143 (100)** | **24978 (100)** | **20 (10-35)** | **5 (1-31)** | **502/1143 (44)** | **28+2 (26+2-30+2)** | **1030 (780-1350)** | **425/1143 (37)** | **43/1143 (4)** | **92/1143 (8)** | **33/1143 (3)** | **125/1143 (11)** | **383/1143 (34)** | **98/1143 (9)** |

**Supplementary Table 2. Patient characteristics** | See Supplementary Table 1 for definitions. *At least one episode of major bleeding/NEC/sepsis/surgery during study follow-up. **At least one day of invasive mechanical ventilation during study follow-up. There was no missing data for the clinical variables in the table.

| Country | Participating centres (n (% of total) | Centres with academic status (n (%)) | Centres that perform NEC surgery (n (%)) | Large centres* (n (%)) |
|---|---|---|---|---|
| Austria | 2 (3) | 2/2 (100) | 2/2 (100) | 1/2 (50) |
| Belgium | 1 (2) | 1/1 (100) | 1/1 (100) | 1/1 (100) |
| Bosnia and Herzegovina | 1 (2) | 1/1 (100) | 1/1 (100) | 1/1 (100) |
| Croatia | 2 (3) | 2/2 (100) | 1/2 (50) | 1/2 (50) |
| Czech Republic | 2 (3) | 2/2 (100) | 1/2 (50) | 2/2 (100) |
| Denmark | 1 (2) | 1/1 (100) | 1/1 (100) | 1/1 (100) |
| France | 6 (9) | 3/6 (50) | 4/6 (67) | 3/6 (50) |
| Germany | 4 (6) | 4/4 (100) | 3/4 (75) | 2/4 (50) |
| Hungary | 3 (5) | 3/3 (100) | 1/3 (33) | 3/3 (100) |
| Ireland | 2 (3) | 2/2 (100) | 0/2 (0) | 2/2 (100) |
| Italy | 8 (13) | 8/8 (100) | 7/8 (88) | 1/8 (13) |
| Netherlands | 3 (5) | 3/3 (100) | 2/3 (67) | 3/3 (100) |
| Norway | 2 (3) | 2/2 (100) | 1/2 (50) | 0/2 (0) |
| Poland | 5 (8) | 4/5 (80) | 3/5 (60) | 1/5 (20) |
| Portugal | 2 (2) | 2/2 (100) | 2/2 (100) | 0/2 (0) |
| Romania | 3 (5) | 2/3 (67) | 0/3 (0) | 3/3 (100) |
| Slovakia | 2 (3) | 2/2 (100) | 1/2 (50) | 0/2 (0) |
| Slovenia | 2 (3) | 2/2 (100) | 1/2 (50) | 1/2 (50) |
| Spain | 6 (9) | 6/6 (100) | 5/6 (83) | 3/6 (50) |
| Sweden | 3 (5) | 3/3 (100) | 2/3 (67) | 2/3 (67) |
| Switzerland | 2 (3) | 2/2 (100) | 2/2 (100) | 1/2 (50) |
| United Kingdom | 2 (3) | 2/2 (100) | 1/2 (50) | 1/2 (50) |
| **Overall** | **64/64 (100%)** | **59/64 (92%)** | **43/64 (67%)** | **33/64 (52%)** |

**Supplemtary Table 3. Participating centres characteristics** | *Large if centre cares for more than 100 preterm infants born below 32 weeks gestation annually.

| Country | **Observed platelet transfusion day prevalence rate*** Per 100 admission days (95% confidence interval) | **Expected platelet transfusion day prevalence rate based on patient-mix**** Per 100 admission days | **Observed/expected ratio***** (95% confidence interval) | **Patient-mix adjusted platelet transfusion day prevalence rate****** Per 100 admission days (95% confidence interval) |
|---|---|---|---|---|
| Austria | 0.06 (0.00-0.95) | 0.42 | 0.15 (0.01-2.26) | 0.05 (0.00-0.78) |
| Belgium | 0.00 | 0.11 | 0.00 | 0.00 |
| Bosnia and Herzegovina | 0.15 (0.02-1.10) | 0.45 | 0.35 (0.5-2.46) | 0.12 (0.02-0.85) |
| Croatia | 1.08 (0.52-2.27) | 0.70 | 1.55 (0.74-3.24) | 0.53 (0.25-1.12) |
| Czech Republic | 0.25 (0.05-1.17) | 0.36 | 0.70 (0.15-3.27) | 0.24 (0.05-1.13) |
| Denmark | 0.65 (0.24-1.73) | 1.12 | 0.58 (0.22-1.55) | 0.20 (0.08-0.53) |
| France | 0.65 (0.32-1.31) | 0.91 | 0.72 (0.36-1.44) | 0.25 (0.12-0.50) |
| Germany | 0.07 (0.00-5.69) | 0.51 | 0.13 (0.00-11.09) | 0.05 (0.00-3.82) |
| Hungary | 0.10 (0.00-11.36) | 1.73 | 0.06 (0.00-6.58) | 0.02 (0.00-2.27) |
| Ireland | 0.22 (0.02-2.50) | 1.05 | 0.21 (0.02-2.38) | 0.07 (0.01-0.82) |
| Italy | 0.60 (0.29-1.22) | 0.78 | 0.77 (0.38-1.57) | 0.27 (0.13-0.54) |
| Netherlands | 0.43 (0.11-1.69) | 0.57 | 0.75 (0.19-2.96) | 0.26 (0.07-1.02) |
| Norway | 1.64 (0.46-5.84) | 1.21 | 1.35 (0.38-4.82) | 0.47 (0.13-1.66) |
| Poland | 0.39 (0.04-3.71) | 0.95 | 0.41 (0.04-3.88) | 0.14 (0.02-1.34) |
| Portugal | 0.00 | 0.50 | 0.00 | 0.00 |
| Romania | 0.15 (0.02-1.03) | 0.94 | 0.15 (0.02-1.10) | 0.05 (0.01-0.38) |
| Slovakia | 0.00 | 0.84 | 0.00 | 0.00 |
| Slovenia | 0.00 | 0.47 | 0.00 | 0.00 |
| Spain | 0.79 (0.41-1.54) | 0.60 | 1.33 (0.68-2.59) | 0.46 (0.23-0.89) |
| Sweden | 0.74 (0.24-2.31) | 0.90 | 0.82 (0.26-2.56) | 0.28 (0.09-0.88) |
| Switzerland | 1.54 (0.38-6.13) | 0.80 | 1.93 (0.48-7.70) | 0.66 (0.17-2.65) |
| United Kingdom | 1.94 (0.16-23.95) | 2.01 | 0.97 (0.08-11.90) | 0.33 (0.03-4.10) |

**Supplementary Table 4. Patient-mix adjusted platelet transfusion day prevalence rates** | *Observed prevalence rates were calculated using random effects Poisson models to pool transfusion day prevalence rates from the individual centres into country subgroup estimates and subsequently to derive the overall estimate. **Expected prevalence rates as predicted based on patient-mix using a logistic regression model which included the following variables: sex assigned at birth, gestational age at birth, birth weight, congenital anomalies, IUGR, major bleeding, NEC, sepsis, mechanical ventilation, surgical procedure, postnatal day. Observed and expected prevalence rates by country, as presented in this table, are also shown graphically in Figure 1 of the main manuscript. ***Observed/expected ratios were calculated by dividing the observed platelet transfusion prevalence rate per country by the expected prevalence rate per country. ****Patient-mix adjusted prevalence rates were calculated by multiplying the country observed/expected ratio with the overall observed prevalence rate (equal to 0.34 platelet transfusion days per 100 admission days).

| Postnatal day | Number of infants at risk | Events | | | Censored |
|---|---|---|---|---|---|
| | | Platelet transfusion | Discharge without platelet transfusion | Death without platelet transfusion | |
| 1 | 468 | 3 | 0 | 7 | 7 |
| 2 | 451 | 2 | 1 | 7 | 10 |
| 3 | 431 | 6 | 2 | 1 | 13 |
| 4 | 409 | 3 | 3 | 3 | 6 |
| 5 | 394 | 5 | 10 | 4 | 12 |
| 6 | 363 | 4 | 2 | 2 | 6 |
| 7 | 349 | 1 | 2 | 2 | 12 |
| 8 | 332 | 2 | 2 | 1 | 9 |
| 9 | 318 | 1 | 6 | 2 | 4 |
| 10 | 305 | 0 | 6 | 0 | 8 |
| 11 | 291 | 2 | 4 | 1 | 8 |
| 12 | 276 | 1 | 4 | 0 | 14 |
| 13 | 257 | 0 | 1 | 0 | 9 |
| 14 | 247 | 0 | 5 | 1 | 6 |
| 15 | 235 | 0 | 2 | 0 | 14 |
| 16 | 219 | 1 | 4 | 0 | 5 |
| 17 | 209 | 0 | 1 | 1 | 14 |
| 18 | 193 | 0 | 1 | 0 | 13 |
| 19 | 179 | 0 | 4 | 0 | 9 |
| 20 | 166 | 1 | 6 | 0 | 7 |
| 21 | 152 | 0 | 5 | 0 | 3 |
| 22 | 144 | 0 | 3 | 1 | 3 |
| 23 | 137 | 0 | 0 | 0 | 4 |
| 24 | 133 | 0 | 3 | 0 | 10 |
| 25 | 120 | 1 | 2 | 0 | 5 |
| 26 | 112 | 0 | 1 | 0 | 6 |
| 27 | 105 | 0 | 0 | 0 | 6 |
| 28 | 99 | 0 | 1 | 0 | 98 |

**Supplementary Table 5. Number at risk table for the cumulative incidence of receiving at least one platelet transfusion during the first 28 postnatal days of life** | Adjusted for the competing risks of death and discharge without platelet transfusion and based on 468/1,143 (40.9%) infants that were followed from birth. Infants were censored if they remained admitted to the NICU at the end of the six week study period or after postnatal day 28.

| Transfusion indication | Platelet count prior to transfusion | |
|---|---|---|
| | Below 25 x10$^9$/L (% of column total) | Equal to or above 25 x10$^9$/L (% of column total) |
| Threshold platelet count | 107 (95) | 53 (54) |
| Active bleeding | 1 (1) | 18 (18) |
| Prevention of major bleeding | 2 (2) | 5 (5) |
| Surgical procedures | 0 (0) | 13 (13) |
| Criticially ill conditions | 3 (3) | 4 (4) |
| Other | 0 (0) | 4 (4) |
| **Total** | 113 (100) | 98 (100) |

**Supplementary Table 6. Transfusion indications stratified by pre-transfusion platelet counts below 25x10$^9$/L or equal to or above 25x10$^9$/L** | Platelet count levels before transfusion (within 24 hours from transfusion) were available in 211 out of 217 (97·2%) transfusions. Platelet counts levels were below 25x10$^9$/L in 113 out of 211 transfusions (53·6%), and equal or above 25x10$^9$/L in 98 out of 211 transfusions (46·4%). Of the 71 (71/1143; 6.2%) infants receiving at least one transfusion, 37 (37/71; 52.1%) patients received two or more platelet transfusions during the study period totaling 146 transfusions. Of these 'follow-up' transfusions, 120 of 146 were given based on threshold (82.2%). Of these 120 threshold 'follow-up' transfusions, 85 (85/119; 71.4%) were given for platelet counts below 25. Platelet count prior transfusion was missing in one of these transfusions (1/120; 0.8%).

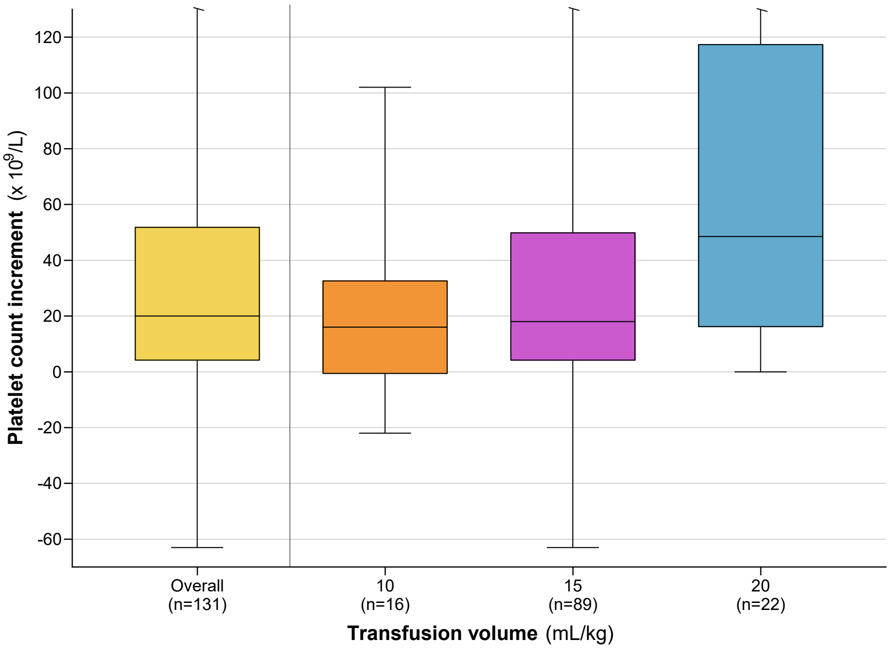


**Supplementary Figure 1. Platelet count transfusion increment stratified per transfusion volume** | Platelet count levels before and after transfusion were available in 131 out of 161 (81·4%) transfusions given based on threshold. Boxplots with fewer than 5 transfusions were omitted from the plot (n=4 transfusions). Platelet count transfusion increment are outside the axis limits in two transfusions at 15 ml/kg and one transfusion at 20ml/kg.

**References**


1. Papile LA, Burstein J, Burstein R, Koffler H. Incidence and evolution of subependymal and intraventricular hemorrhage: a study of infants with birth weights less than 1,500 gm. *J Pediatr* 1978; **92**(4): 529-34.

2. Bell MJ. Neonatal necrotizing enterocolitis. *The New England journal of medicine* 1978; **298**(5): 281-2.

3. Hoftiezer L, Hof MHP, Dijs-Elsinga J, Hogeveen M, Hukkelhoven C, van Lingen RA. From population reference to national standard: new and improved birthweight charts. *Am J Obstet Gynecol* 2019; **220**(4): 383.e1-.e17.

# INSPIRE-study
## **(I**nternational **N**eonatal tran**S**fusion **P**o**I**nt p**RE**valence)

*Research Protocol*

**PROTOCOL TITLE** 'INSPIRE-study: *International Neonatal tranSfusion PoInt pREvalence*'

| | |
|---|---|
| **Protocol ID** | G21.207 |
| **Short title** | INSPIRE-study |
| **EudraCT number** | Not applicable |
| **Version** | 3.1 |
| **Date** | June 6, 2023 |
| **Coordinating investigator/project leader** | N.A.M. Houben, PhD student<br>Sanquin Blood Supply Foundation / Leiden University Medical Center<br>Plesmanlaan 125 / Albinusdreef 2<br>1066 CX Amsterdam / 2333 ZA Leiden, The Netherlands<br>n.a.m.houben@lumc.nl<br>Tel. +31 (0)6-20985851 |
| **Principal investigator(s)** | Dr. S.F. Fustolo-Gunnink<br>Sanquin Blood Supply Foundation<br>Plesmanlaan 125<br>1066 CX Amsterdam, The Netherlands<br>s.f.gunnink@amsterdamumc.nl<br>Tel. +31 (0)20-5123000<br><br>Prof. Dr. E. Lopriore<br>Department of Paediatrics<br>Leiden University Medical Center<br>Albinusdreef 2<br>2333 ZA Leiden, The Netherlands<br>e.lopriore@lumc.nl<br>Tel. +31 (0)71-5262965<br><br>Prof. Dr. C.J. Fijnvandraat<br>Department of Paediatric Haematology<br>Amsterdam UMC<br>Meibergdreef 9<br>1105 AZ Amsterdam<br>c.j.fijnvandraat@amsterdamumc.nl<br>Tel. +31 (0)20-5662727<br><br>Prof. Dr. J.G. van der Bom<br>Department of Epidemiology<br>Leiden University Medical Center<br>Albinusdreef 2<br>2333 ZA Leiden, The Netherlands<br>j.g.vanderbom@lumc.nl<br>Tel. +31 (0)71-5685053<br><br>Prof. Dr. Christof Dame<br>Department of Neonatology<br>Charité - University Hospital Berlin<br>Augustenburger Platz 1<br>13353 Berlin, Germany |

| | |
|---|---|
| | christof.dame@charite.de<br>Tel. +49 (0)30-450559006<br><br>Prof. Charles C Roehr<br>Newborn Services<br>Southmead Hospital, North Bristol Trust<br>Southmead Road, Bristol BS10 5NB<br>Bristol, UK<br>charles.roehr@bristol.ac.uk<br><br>Dr. Emöke Deschmann<br>Karolinska Institute<br>Solna, Sweden<br><br>emoke.deschmann@regionstockholm.se<br><br>Prof. Dr. Simon Stanworth<br>NHS Blood and Transplant (NHSBT) and University of Oxford<br>London, United Kingdom<br>simon.stanworth@nhsbt.nhs.uk<br><br>Dr. Helen New<br>NHSBT and Imperial College London<br>London, United Kingdom<br>helen.new@nhsbt.nhs.uk |
| **National coordinators** | **Austria**<br>Dr. Francesco Cardona<br>Medical University Vienna<br>Vienna, Austria<br><br>**Belgium**<br>Prof. Anne Debeer<br>UZ Leuven<br>Leuven, Belgium<br><br>**Bosnia and Herzegovina**<br>Prof. dr. Emina Hadžimuratović<br>University Medical Center Sarajevo<br>Sarajevo, Bosnia and Herzegovina<br><br>**Croatia**<br>Prof. dr. Ruza Grizelj<br>University Hospital Center Zagreb<br>Zagreb, Croatia<br><br>**Czech Republic**<br>Dr. Jan Malý<br>University Hospital Hradec Králové<br>Hradec Králové, Czech Republic<br><br>**Denmark**<br>Christian Heiring |

| | |
|---|---|
| | Neonatalklinikken Rigshospitalet<br>Copenhagen, Denmark<br><br>**France**<br>Prof. Dr. Alain Beuchée<br>CHU de Rennes<br>Rennes, France<br><br>**Germany**<br>Prof. Dr. Christof Dame<br>Charité - University Hospital Berlin<br>Berlin, Germany<br><br>**Hungary**<br>Dr. Miklós Szabó<br>Semmelweis University<br>Budapest, Hungary<br><br>**Ireland**<br>Dr. Carmel Maria Moore<br>University College Dublin – The National Maternity Hospital<br>Dublin, Ireland<br><br>**Italy**<br>Prof. dr. Stefano Ghirardello<br>NICU Fondazione IRCCS Policlinico San Matteo<br>Pavia, Italy<br><br>**Norway**<br>Dr. Kristin Brække<br>Oslo University Hospital, Ullevål<br>Oslo, Norway<br><br>**Poland**<br>Dr. Tomasz Szczapa<br>Poznań University of Medical Sciences<br>Poznan, Poland<br><br>**Portugal**<br>Dr. Sara Domingues<br>Centro Materno Infantil do Norte<br>Porto, Portugal.<br><br>**Romania**<br>Prof. dr. Gabriela Zaharie<br>University of Medicine and Pharmacy Iuliu Hatieganu Cluj<br>Cluj Napoca, Romania<br><br>**Slovakia**<br>Dr. Katarina Matasova<br>Martin University Hospital<br>Martin, Slovakia |

| | |
|---|---|
| | **Slovenia**<br>Dr. Jana Lozar Krivec<br>University Medical Center Ljubljana<br>Ljubljana, Slovenia<br><br>**Spain**<br>Dr. Marta Aguar Carrascosa<br>La Fe University Hospital<br>Valencia, Spain<br><br>**Sweden**<br>Dr. Emöke Deschmann<br>Karolinska Institute<br>Solna, Sweden<br><br>**Switzerland**<br>Dr. Tobias Muehlbacher<br>University Hospital Zurich<br>Zurich, Switzerland<br><br>**The Netherlands**<br>Prof. Dr. Enrico Lopriore<br>Leiden University Medical Center<br>Leiden, The Netherlands<br><br>**United Kingdom**<br>Prof. Charles Roehr<br>Southmead Hospital, North Bristol Trust<br>Bristol, UK |
| **Sponsor** | Leiden University Medical Center<br>Albinusdreef 2<br>2333 ZA Leiden, The Netherlands |
| **Subsidizing parties** | Sanquin Blood Supply Foundation<br>Clinical Transfusion Research Dept.<br>Plesmanlaan 125<br>1066 CX Amsterdam, The Netherlands<br><br>European Society for Pediatric Research (ESPR)<br>Rue de Sablieres 5<br>1242 Satigny, Switzerland<br><br>European Blood Alliance (EBA)<br>Plesmanlaan 125<br>1066 CX Amsterdam, The Netherlands |
| **Advisory board** | Dr. O. Karam<br>Division of Pediatric Critical Care Medicine<br>Children's Hospital of Richmond at VCU<br>Richmond, United States<br>oliver.karam@vcuhealth.org |

| | |
|---|---|
| | Dr. M.E. Nellis<br>Pediatric Critical Care Medicine<br>NY Presbyterian Hospital and Weill Cornell Medicine<br>New York, United States<br>man9026@med.cornell.edu<br><br>Dr. M.C. Sola-Visner<br>Division of Newborn Medicine<br>Boston Children's Hospital and Harvard Medical School<br>Boston, United States<br>martha.sola-visner@childrens.harvard.edu<br><br>Dr. A. Keir<br>South Australian Health and Medical Research Institute and Adelaide Medical School<br>North Adelaide, Australia<br>amy.keir@adelaide.edu.au<br><br>Prof. N.L.C. Luban<br>Children's National Hospital and The George Washington University School of Medicine and Health Sciences<br>Washington, United States<br>nluban@childrensnational.org |

## PROTOCOL SIGNATURE SHEET

| Name | Signature | Date |
|---|---|---|
| **Head of Division:**<br><br>Prof. Dr. E. Lopriore<br>Division of Neonatology,<br>Department of Paediatrics<br>Leiden University Medical Center<br>Albinusdreef 2<br>2333 ZA Leiden, The Netherlands<br>e.lopriore@lumc.nl<br>Tel. +31 (0)71-5262965 | | *11-03-2022* |
| **Principal Investigator:**<br><br>Dr. S.F. Fustolo-Gunnink<br>Sanquin Blood Supply Foundation<br>Plesmanlaan 125<br>1066 CX Amsterdam, The Netherlands<br>s.f.gunnink@amsterdamumc.nl<br>Tel. +31 (0)20-5123000 | | *11-03-2022* |
| **Coordinating Investigator:**<br><br>N.A.M. Houben, PhD student<br>Sanquin Blood Supply Foundation / Leiden University Medical Center<br>Plesmanlaan 125 / Albinusdreef 2<br>1066 CX Amsterdam / 2333 ZA Leiden, The Netherlands<br>n.a.m.houben@lumc.nl<br>Tel. +31 (0)6-20985851 | | *11-03-2022* |

## PROTOCOL AGREEMENT PAGE

I agree to conduct the Clinical Study in accordance with the current protocol and comply with its requirements, subject to ethical and safety considerations.

Institute ............................................

Local Investigator Name ............................................

Local Investigator Signature ............................................

Date ............................................

## TABLE OF CONTENTS

1. SUMMARY ..... 11
2. INTRODUCTION AND RATIONALE ..... 12
3. OBJECTIVES ..... 14
3.1 Primary Objective ..... 14
3.2 Secondary Objectives ..... 14
4. STUDY DESIGN ..... 15
4.1 Study design ..... 15
4.2 Duration ..... 15
4.3 Setting ..... 15
5. STUDY POPULATION ..... 16
5.1 Population (base) ..... 16
5.2 Inclusion criteria ..... 16
5.3 Exclusion criteria ..... 16
5.4 Sample size calculation ..... 16
6. METHODS ..... 18
6.1 Main study endpoint ..... 18
6.2 Secondary study endpoints ..... 18
6.3 Study procedures ..... 20
7. SAFETY REPORTING ..... 21
8 STATISTICAL ANALYSIS ..... 22
9. ETHICAL CONSIDERATIONS ..... 23
9.1 Regulation statement ..... 23
9.2 Consent ..... 23
10. ADMINISTRATIVE ASPECTS, MONITORING AND PUBLICATION ..... 25
10.1 Handling and storage of data and documents ..... 25
10.2 Monitoring and Quality Assurance ..... 25
10.3 Amendments ..... 25
10.4 End of study report ..... 25
10.5 Public disclosure and publication policy ..... 25
11. REFERENCES ..... 26

## LIST OF ABBREVIATIONS AND RELEVANT DEFINITIONS

| | |
|---|---|
| **(S)AE** | (Serious) Adverse Event |
| **ABR** | General Assessment and Registration form (ABR form), the application form that is required for submission to the accredited Ethics Committee |
| **CCMO** | Central Committee on Research Involving Human Subjects |
| **CV** | Curriculum Vitae |
| **DSMB** | Data Safety Monitoring Board |
| **ECMO** | Extracorporeal Membrane Oxygenation |
| **EU** | European Union |
| **GCP** | Good Clinical Practice |
| **GDPR** | General Data Protection Regulation |
| **IC** | Informed Consent |
| **IRB** | Institutional Review Board |
| **METC** | Medical Research Ethics Committee |
| **NEC** | Necrotizing EnteroColitis |
| **NICU** | Neonatal Intensive Care Unit |
| **NTN** | Neonatal Transfusion Network |
| **RBC** | Red Blood Cell |
| **Sponsor** | The sponsor is the party that commissions the subsidizing or performance of the research, for example a pharmaceutical company, academic hospital, scientific subsidizing, or investigator. A party that provides funding for a study but does not commission it is not regarded as the sponsor but referred to as a subsidizing party. |

# 1. SUMMARY

**Rationale:** Premature neonates are highly transfused patients, though robust evidence supporting neonatal transfusion practice is scarce. Two randomized controlled trials (RCTs) were recently published, indicating no benefit in long-term outcomes when using liberal (high) thresholds for red blood cell (RBC) transfusions. Another RCT, comparing a high and low platelet transfusion threshold, even reported evidence that liberal transfusion treatment (high thresholds) can cause harm. There are no international neonatal transfusion guidelines that have been implemented by Europe as a whole, resulting in significant variation in transfusion practice within Europe. Detailed contemporary data on neonatal transfusion practice in Europe, including neonatal component specifications, are lacking. This point prevalence study will provide a picture of current neonatal transfusion practices within Europe, which can be used to improve practice, promote adherence to evidence-based transfusion guidelines, and inform future randomized controlled trials.

**Main objective**: To describe the prevalence, indications, adverse effects, and component specifications of RBC, platelet, and plasma transfusions in preterm neonates. Additionally, to describe the use of local or national guidelines and the evidence-basedness of transfusion practices in preterm neonates.

**Study design:** Prospective, European, multicenter, observational point prevalence study.

**Study population:** Neonates with a gestational age of less than 32 weeks at birth who are admitted to a participating tertiary level Neonatal Intensive Care Unit (NICU).

**Expected results:** This study will identify current neonatal transfusion practices that can be improved, and areas with substantial clinical variation which can be targeted in future clinical trials. The resulting data may help reduce unnecessary transfusions through increased awareness of the proper use of transfusions in this vulnerable patient population. This may eventually lead to a reduction in the number of adverse events, lower costs, optimal allocation of donor blood, and ultimately, better long-term neonatal outcomes.

## 2. INTRODUCTION AND RATIONALE

**Every year, approximately 66,000 very premature babies in Europe receive one or more blood transfusions. (1) Despite the high frequency of transfusions in this population, the efficacy and safety of many of these transfusions are not known, as the number of appropriate trials in this population is limited.** The blood components most frequently transfused to babies are red blood cells (RBCs), platelets and plasma. Four randomized controlled trials assessing RBC transfusion thresholds have been published, of which two were published in 2020. (2-5) Two trials assessed platelet transfusions and only one trial assessed fresh frozen plasma (FFP) transfusions. (1, 6-8) As a broad generalization, the findings of these studies do not support the use of liberal transfusion policies, but data on optimal thresholds, dosing and product specifications are still lacking.

**Recent studies have shown that many neonatal transfusions may be redundant.** Redundant transfusions are transfusions given at thresholds or indications that are not supported by existing evidence and are therefore not expected to convey benefit to the neonate. Redundant transfusions are problematic because they expose babies to unnecessary risks, lead to increased costs and undue burden on health care facilities and donor blood availability. The ETNNO-trial (‘Effects of Liberal vs Restrictive Transfusion Thresholds on Survival and Neurocognitive Outcomes in Extremely Low-Birth-Weight Infants’) and TOP-trial (‘Transfusion Of Prematures’), assessing liberal versus restrictive RBC transfusion thresholds, both found that restrictive thresholds were non-inferior to liberal thresholds. The PlaNeT-2/MATISSE platelet transfusion trial (‘Platelets for Neonatal Transfusion - study 2 / MAnaging Thrombocytopenia in a Special Subgroup: nEonates’) showed that a 25x$10^9$/L platelet count threshold was superior to a 50x$10^9$/L threshold in preterm infants. (2, 3) In short, all three studies support restrictive transfusion strategies. Despite these findings, our recent European neonatal transfusion survey, which included 341 NICU’s from 18 countries, showed 53% of platelet transfusions given at a threshold >25x$10^9$/L The interquartile ranges (IQR) for haemoglobin thresholds in different clinical scenarios varied between 6-20 g/L. (9)

**Recent studies have also shown that neonatal transfusions may cause side effects or even direct harm**. Transfusion-associated side effects in neonates are poorly defined, and therefore likely to be under-recognized. Despite this likely under-recognition, data from national hemovigilance systems in the Netherlands and the UK suggest that neonates are at much higher risk of transfusion-associated adverse events compared to children or adults. (10) More importantly, there is evidence that transfusions may cause direct harm. The PlaNeT-2/MATISSE trial, comparing a high (50x$10^9$/L) and low (25x$10^9$/L) platelet transfusion threshold, showed unexpected higher rates of mortality and/or severe bleeding, in the higher threshold (liberal transfusion) study group. (7) The mechanisms of this apparent platelet transfusion-associated harm have yet to be elucidated in detail but indicate that the potential benefits of transfusions must be carefully weighed against the risk of transfusion-related harm.

**There are no neonatal transfusion guidelines that have been implemented by Europe as a whole, and there is significant variation in transfusion practice within Europe.** Two national guidelines specific to neonatal transfusions have been published in peer-reviewed journals, but whether their recommendations apply to the whole of Europe remains to be determined. (11, 12) It is unknown whether other countries or individual NICU’s have national or local guidelines and what these guidelines recommend. To describe neonatal transfusion practices in Europe, we have recently performed a large European neonatal transfusion survey in which 18 countries participated. We analysed data from 341 NICU’s. The results of this survey suggest that a substantial number of transfusions may be redundant, as described previously. The results also showed wide variation in thresholds, even within relatively well-defined patient groups. For example, the interquartile range for red

blood cell transfusion haemoglobin thresholds for stable preterm infants of less than 32 weeks gestational age in the first week of life was 80-100 g/L, with 25% of centers using more extreme thresholds ranging from 50-140 g/L. Platelet transfusion thresholds for stable, non-bleeding infants with a gestational age of less than 28 weeks at birth, varied between no prophylaxis up to transfusion at a platelet count of $100x10^9$/L, with an IQR of 20-$47x10^9$/L. Volumes and rates of transfusion also varied substantially. These results are in line with older surveys and a recent report from the USA showing similarly variable transfusion patterns. (13-16)

**A prospective point prevalence study is timely and will provide crucial data for new randomized trials and implementation projects.** The neonatal transfusion survey was a crucial first step to improve neonatal transfusion practices but needs to be followed up by a prospective study for several reasons. The survey targeted only one neonatologist per NICU, which did not allow for assessment of within unit variation in practice. More importantly, we need exact data about transfusion prevalence, indications, and specifications to be able to set up new randomized controlled trials and develop effective implementation strategies. We did not collect data on component specification or on use of local or national transfusion guidelines. And lastly, a known limitation of surveys is that reported behaviour may differ from actual behaviour, whether intentionally or unintentionally. Therefore, a prospective international observational study is warranted.

**The INSPIRE will be performed by the Neonatal Transfusion Network (NTN), an international, interdisciplinary neonatal transfusion research network**. The NTN aims to improve current practices and generate more evidence for neonatal transfusion medicine. As of January 2021, approximately 100 participants from 31 countries are represented in this network. The European Blood Alliance (EBA), the European Society for Pediatric Research (ESPR), the International Haemovigilance Network (IHN) and the European Foundation for the Care of the Newborn Infant (EFCNI) have endorsed the NTN. As all stakeholders, including neonatologists, haematologists, epidemiologists, as well as parent representatives and blood bank agents, collaborate within our international NTN, the results of this study can directly be translated into practice change and may thus substantially impact the quality of neonatal transfusion medicine in Europe and worldwide.

To summarize, neonates receive blood transfusions even though they might not be effective or could be harmful, as current transfusion guidelines are not supported by sufficient evidence and existing evidence has not yet been incorporated into clinical practice. Whilst the neonatal population may seem small, over the next 20 years, approximately 1 million infants in Europe will receive a transfusion while being a premature newborn. The potential short- and long-term effects of these transfusions should therefore not be underestimated. Recent consensus meetings and scientific reviews underline the need for high quality, global epidemiologic data as a first step towards improving neonatal transfusion medicine. (1, 17-19) These data will help to improve practice, develop research protocols, and inform guideline writing. **We therefore aim to perform a European point prevalence study (INSPIRE), which will provide high quality multinational epidemiologic data that can be used to improve neonatal transfusion medicine in Europe.**

# 3. OBJECTIVES

To describe the prevalence, indications, adverse effects, and component specifications of transfusions among preterm neonates admitted to tertiary level NICUs in Europe. Additionally, to describe the use of guidelines and the evidence-basedness of transfusion practices in very preterm neonates.

## 3.1 Primary Objective

To describe the prevalence of RBC, platelet, and plasma (FFP and cryoprecipitate) transfusions in very preterm neonates with a gestational age less than 32 weeks at birth admitted to a tertiary level NICU.

## 3.2 Secondary Objectives

To describe the variations in prevalence, the indications for transfusion, duration, volume, rate, adverse effects and component specifications of the prescribed RBC, platelet, or plasma transfusions. Additionally, to assess the number of transfusions per transfused neonate, the proportion of neonates who received at least one transfusion, the incidence of receiving at least one transfusion. Furthermore, to describe use of guidelines and the evidence-basedness of transfusion practices in preterm neonates with a gestational age less than 32 weeks at birth. Lastly, to assess the use of transfusions with blood products other than RBC, platelet, and plasma or agents that promote or reduce coagulation.

# 4. STUDY DESIGN

## 4.1 Study design

International, multicenter, prospective, observational point prevalence study.

## 4.2 Duration

Data will be collected over a one-year period. During this year, each participating NICU will collect data during a six-week period in which they will screen all 7 days of each week.

## 4.3 Setting

Data collection will take place in tertiary level NICUs in Europe, a minimum of 61 NICUs must be recruited to reach our sample size (see section 5.4). A tertiary level NICU is defined as a hospital NICU organized with personnel and equipment to provide continuous life support and comprehensive care for extremely high-risk newborn infants with a gestational age of less than 32 weeks, and those with complex and critical illness. (20)

# 5. STUDY POPULATION

## 5.1 Population (base)

Approximately 700,000 neonates are born prematurely each year in Europe, of whom about 16% are born very premature at a gestational age of less than 32 weeks. (21) Within this population, neonates will be included based on inclusion and exclusion criteria defined below.

## 5.2 Inclusion criteria

A potential subject who meets both of the following criteria will be included in this study:

1. Admission to a participating tertiary level NICU (including outborn neonates or neonates readmitted to the NICU)
2. Gestational age at birth below 32 weeks.
   Note: gestational age at admission can be >32 weeks, however the postmenstrual age (PMA; gestational age + chronological age) at inclusion cannot exceed 44+0 weeks.

## 5.3 Exclusion criteria

No exclusion criteria.

## 5.4 Sample size calculation

*5.4.1 Sample size calculation*

There are several ways to describe transfusion practices, such as describing the proportion of neonates receiving any number of transfusions, the number of neonates receiving a first transfusion (incidence) or describing the total number of transfusions given per patient-time period (prevalence rate). We have chosen the prevalence rate as our primary outcome, because this takes into account variations in duration of follow up and variations in the total number of transfusions neonates receive.

We will calculate the prevalence rates by dividing the number of transfusions of the respective transfusion type by the total sum of neonate study days. We performed sample size calculations for RBC, platelet, and plasma transfusion prevalence rates, estimating the sample size necessary to ensure that the confidence interval for a prevalence is of a predetermined width. Based on data from previously conducted studies, we expect the approximate rates of RBC, platelet, and plasma transfusions to be 2.0 (1.3-2.6), 0.9 (0.6-0.12) and 0.4 (0.2-0.6) transfusions per 100 study days, respectively. (2, 3, 7, 16, 22-24) We aim to estimate the prevalence rates with sufficient certainty, which we have defined as rates within +/-0.5 for RBC, +/- 0.2 for platelet, and +/-0.1 for plasma transfusions per 100 study days. This corresponds to half of the width of the confidence interval for each transfusion type found in the literature. To achieve this level of certainty, we have to obtain data on 3152, 6833, and 15366 study days for RBC, platelet and plasma transfusions, respectively.

We expect that each participating NICU will contribute on average 252 patient days. This number is based on the length of the data collection period (42 days), the estimated percentage of eligible NICU admissions with gestational age <32 weeks (30%) and the estimated number of beds per unit (20, unpublished data obtained within the NTN). We therefore expect we need to recruit at least 61 (15366/252) NICUs to reach our sample size. As we will collect data in a large geographical area, we aim to recruit NICUs in at least 10 countries and recruit at least 10% of NICUs within each country or at least 2 centers per country, whichever is greater, even if this will result in a sample size higher than 61. The

number of centers included per country will be proportional to the population size of the country compared to that of all other included countries.

Details of the interim analysis will be included in the statistical analysis plan prior to the interim analysis. If needed, results of the interim analysis can lead to implementation one of two measures: extending de data collection period or recruiting additional centers. Any decisions following the interim analysis will be made in close consultation with the national coordinators.

# 6. METHODS

## 6.1 Main study endpoint

The primary outcome of this study is the prevalence of RBC, platelet, and plasma (FFP and cryoprecipitate) transfusions in neonates with a gestational age of less than 32 weeks at birth, admitted to a tertiary level NICU. The prevalence is calculated by dividing the number of transfusions of the respective transfusion type by the total sum of neonate study days.

A transfusion is registered if administered to a neonate during the study period. Transfusions issued by the blood bank but not administered will not be registered.

To calculate the total sum of neonate study days, each center will register date and time of start and end of study of all eligible neonates during the center's data collection period. For neonates already admitted at the start of the study, the start of study will be the moment the data collection period starts. For all other infants, start of study equals time of admission. These data will allow us to calculate the exact number of study days for each individual neonate in the study.

To describe the study population, we will collect NICU characteristics (e.g. unit size) and neonatal baseline characteristics (e.g. gestational age).

## 6.2 Secondary study endpoints

*6.2.1 Number of transfusions per transfused neonate*
We will assess the number of transfusions per neonate for RBC transfused neonates, platelet transfused neonates and plasma transfused neonates.

*6.2.2 Proportion of neonates who received at least one transfusion*
We will calculate the proportion of neonates who received at least one transfusion during the study period, per transfusion type.

6.2.3 Incidence of receiving at least one transfusion
We will calculate the incidence of receiving at least one transfusion, both for any transfusion as per transfusion type. The incidence is calculated by neonates receiving at least one transfusion by the total sum of neonate study days.

*6.2.4 Variations in prevalence*
We will describe the variations in prevalence rates of RBC, platelet, and plasma transfusions. Data analysis will be predominantly descriptive, comparing both between participating countries and between different type of NICUs. Types of NICU's will be defined based on unit size and whether surgical procedures are being performed.

*6.2.5 Indication for transfusion*
We will collect the primary indication for which the transfusion is prescribed by the treating physician. If applicable, we will also register the secondary and tertiary indication, as clinicians may consider multiple factors when deciding to give a transfusion. For each transfusion, the prescribing physician must choose from a list of predefined indications (up to three options can be selected, ranked primary to tertiary indication). The primary indication is the indication that has the most weight in the decision to prescribe a transfusion. If the indication for transfusion is registered by someone other than the prescribing physician, the prescribing physician must confirm the correctness of these data.

*6.2.6. Duration, volume, and transfusion rate*

We will assess the duration of transfusion, transfusion volume, and transfusion rate of the prescribed RBC, platelet, and plasma transfusions. We will describe the variations in duration of transfusion, transfusion volume, and transfusion rate.

*6.2.7 Guideline use*

We will collect data on whether centers have guidelines in place regarding RBC, platelet, and plasma transfusion for neonates. Following the implementation model by Wensing and Grol, we aim to gather information on the implementation of transfusion guidelines. (25) Additionally, we will ask centers with established guidelines to provide us with the guidelines in place at the start of data collection. We will analyse how many of the RBC and platelet guidelines have already incorporated the results of the recent clinical trials. We will categorize the existing RBC guidelines into "TOP and/or ETTNO incorporated" and "TOP and/or ETTNO not incorporated".(2, 3) We will categorize the existing platelet guidelines into "PlaNeT-2/MATISSE incorporated" and "PlaNeT-2/MATISSE not incorporated". We will assess to what extent centers follow their own local and/or national guidelines. (7)

*6.2.8 Evidence-basedness of practice*

Before the start of data collection, we will collaborate with various experts to define what we view as high quality evidence regarding RBC and platelet transfusion practices in neonates, using the best available evidence including the TOP, ETTNO, and PlaNeT-2/MATISSE trials. (2, 3, 7) We will assess if the RBC and platelet transfusions were prescribed following the best available evidence, by categorizing the prescribed transfusions into different levels of certainty in evidence. We will assign a panel review by three 'blinded' experts to discuss cases where there may be ambiguity on the certainty of evidence to support transfusion practice. Transfusion events in clinical scenarios that were not addressed in randomized trials will not be assessed.

*6.2.9 Transfusion-associated adverse effects*

In the absence of clear definitions of transfusion-associated side effects in neonates, we will ask participating centers to register any perceived transfusion-associated adverse effects if the local investigators consider the adverse event to be potentially associated with the preceding transfusion. With this we hope to gain insight into what adverse effects neonatologists identify in clinical practice, despite the lack of well-defined descriptions.

*6.2.10 Component specifications*

We will perform an online survey among transfusion experts in which we explore variations in transfusion component characteristics. We chose this strategy because neonatologists may not be aware of all relevant component specifications. The survey will be sent to transfusion experts in all countries participating in this study during the data collection period. We will record the blood banks from which each participating NICU receives their blood products, which will allow us to link the NICU clinical data to the component specifications reported by the transfusion specialists. We aim to include all blood banks that provide blood products to participating NICUs in our survey.

*6.2.11 Transfusion with blood products other than RBC, platelet, and plasma or agents that promote or reduce coagulation.*

We will collect data on transfusions of blood product other than RBC, platelet, and plasma transfusions or agents that promote or reduce coagulation, such as erythropoietin. We will describe these treatments and the indications for which they were prescribed.

### 6.3 Study procedures

*Participating sites*
The study aims to include a broad range of European countries. Each country will be represented by a national coordinator for this study, who will be responsible for recruiting centers and coordinating ethical procedures in collaboration with the study coordinator. Based on the number of European countries participating, we will distribute the required 61 NICUs proportionally to the population size of the country, determining the minimum number of centers each country has to include. In cooperation with the national coordinators, we aim to achieve, where possible, a representative sample for their country, based on one or more of the following factors: unit size, non-academic vs academic centers, surgical vs non-surgical centers. Each site will have a local investigator who is responsible for the study in their NICU, including the coordination of the ethical procedure and data collection.

*Pilot*
We aim to perform a pilot study in 5 centers to check feasibility of and the workload for our study protocol. This can inform us on the workload on collecting and entering patient data for the study. Additionally, it can help identify possible problems that participating centers may encounter during the entry. There will be no data sharing of patient data in the pilot. If needed, modifications to the protocol and/or database will be made based on the results of the pilot study.

*Parental involvement*
The Neonatal Transfusion Network strives to achieve parental involvement in neonatal transfusion medicine studies. Preceding the finalization of the database, the NTN will establish an international parental advisory board, in collaboration with the European Foundation for the Care of Newborn Infants (EFCNI). This board will be invited to provide input for the study protocol. If needed, modifications to the protocol and/or database will be made based on this feedback.

*Allocation of data collection periods*
Data will be collected over a one-year period to account. Study sites will have the opportunity to choose a preferred start date from a limited number of options, aiming to start within 4 months after ethical approval by their local institutional review board and signing of the data sharing agreement (DSA), allowing centers to select the option that is most convenient for them. We aim to start data collection in the spring of 2022.

*Data collection*
Each site will collect data during a six-week period. As not to bias against sites with differing working days, the centers will screen all 7 days of the assigned weeks, using an online database. Data will be collected by study personnel, which may include research nurses, data managers, medical students, and PhD students, under supervision of the local investigator. We will provide online data entry training opportunities. Data will be collected from the hospital's written or electronic patient record files, recorded imaging reports, hospital blood bank records, and nurses' records. Study data will be entered into the electronic data capture tool Castor. Data collection ends at the end of the six-week period.

*Outborn or readmitted infants*
Infants that are outborn or readmitted to the unit will be included in the study. Readmitted neonates will not receive a new study number, but data collection will continue under the study number they received during their first admission. Data collection for outborn infants will be identical to data collection for inborn infants, as we will not collect data from the period before admission.

## 7. SAFETY REPORTING

The International Neonatal Transfusion Point Prevalence Study is an observational study, we therefore consider reporting of adverse events or serious adverse events not applicable for this study.

# 8 STATISTICAL ANALYSIS

A full data analysis plan will be drawn up before the start of the data analysis. Data analysis will be predominantly descriptive, with primary results provided of all countries combined, as well as data on individual countries. We will not publish non-anonymised data of individual centers. To calculate the overall prevalence rate for each transfusion type, we will first determine the prevalence rate per country, as some countries may include more than the required number of NICU's based on their population size. The prevalence rate per country will be calculated by dividing the number of transfusions of the respective transfusion type prescribed in the country by the total sum of neonate study days of the country. Thereafter, the overall prevalence will be determined by calculating the average in proportion to population size.

We will perform relevant subgroup and sensitivity analyses, including subgroup analyses for gestational age, birthweight, neonates with congenital malformations, neonates who underwent surgery, and subgroups based on center characteristics. Sensitivity analyses will at a minimum include an analysis excluding neonates with congenital malformations and neonates with gestational age <24 weeks. Missing data will be handled using simple imputation or multiple imputations where appropriate. Dependent on the distribution of the variables, the results will be described as proportions (confidence intervals), as means (standard deviations (SD)) or as medians (interquartile range (IQR)). All tests will be two-sided, with an α level of 0.05.

# 9. ETHICAL CONSIDERATIONS

## 9.1 Regulation statement

This study will be conducted according to the principles of the Declaration of Helsinki (64th WMA General Assembly, October 2013) and the General Data Protection Regulation (GDPR).

## 9.2 Consent

We will submit the study protocol to the institutional review boards of the coordinating and participating centers for ethical approval. Our application will be handled according to the local and/or national rules and procedures that apply in the participating foreign centers. Each site will be responsible for obtaining ethical consent if required by their institution, in collaboration with the study coordinator.

We will request waiver of consent, which means parents do not have to give consent for their child's data to be used in this study. The decision to apply for waiver of consent for the INSPIRE-study was made considering the trade-offs, where we feel that these advantages are considered to outweigh the disadvantages of approaching all parents individually for consent. Our rationale is that we aim to avoid more burden for parents with a waiver of consent and that the validity and generalisability of our study would be threatened by selection bias if waiver of consent is not granted.

A. **Avoiding selection bias**
   Consent bias, a type of selection bias, can occur when neonates whose parents do not give consent differ from neonates whose parents do. A recent study by Weiss et al. assessed the parental factors associated with their participation in neonatal research. (26) They found that both parents with lower socioeconomic status and Black parents more frequently declined consent for their infant's participation. Previous studies have shown that neonatal outcomes of both infants from parents with lower incomes as well as Black infants are worse. (27-33) Additionally, two studies comparing the population representation in neonatal clinical research found higher (severe) morbidity and mortality rates among the non-enrolled preterm infants compared to enrolled preterm infants. (34, 35) High transfusion rates have previously been described in premature neonates with severe comorbidity. (36) If informed consent is required, certain groups that are expected to receive a substantial proportion of transfusions would thus be systematically underrepresented in our study. This will have important implications for the generalisability of the findings of our study to the wider neonatal population. More importantly, it will perpetuate existing disparities in clinical outcomes between these groups as they do not benefit from the research. As we will use our findings to inform future studies, the biased results would make it impossible to provide meaningful guidance for the design of randomized controlled trials and quality improvements projects. Consent bias thus has serious implications for future research, as these studies should benefit all premature neonates. In conclusion, consent bias threatens the validity, relevance, and generalizability of our study.

B. **Avoiding more burden for parents**
   For many parents, the first days of their newborn's stay in the NICU are very stressful and they may feel overwhelmed by the huge amount of information they receive. However, as most neonates receive blood transfusions during the first week of life, it is important to be able to start data collection immediately after birth to avoid missing transfusion events. (22, 37, 38) During these early days, the additional information that parents may receive through an informed consent procedure for their child's participation in clinical research can contribute to this overload. The available

research assessing this burden in the NICU setting is very limited to our knowledge, so we have also identified several pivotal studies in a similar setting, the Pediatric Intensive Care Unit (PICU). A study by Hodson et al. found that 52% of the parents of children in the PICU felt overwhelmed when approached for their consent to non- or minimal risk observational studies. (39) Two other studies assessing consent for participation in clinical research in the PICU both found that parents most commonly described feeling too stressed or overwhelmed to consider participation as the reason for withholding consent. (40, 41) As parents of severely ill neonates are likely to be the most stressed and therefore less likely to consent, this may also contribute to selection bias described above. Moreover, a study by Rich and Katheria, examining parents' perceptions of a waiver of consent for participation in a neonatal clinical trial, found that the majority of the parents felt positive or strongly positive about their infant's participation in the study. (42) Given the emotional burden experienced by parents during their child's NICU stay and that there are no or minimal risks associated with participation in our study, we feel that waiver of consent is the most fitting approach.

We do, however, feel that it is important to inform parents about the participation of their child in the study. Together with the European Foundation for the Care of Newborn Infants (EFCNI), we have drafted an information letter to inform parents about the study and provide them the opportunity to receive the study results. This letter can be given to the parents at a time when it is more convenient for them and they no longer feel overwhelmed. Additionally, the EFCNI has expressed its support for our request for a waiver of consent (see document '*K6.2 Support letter EFCNI 5-10-2021*').

If a waiver is not granted, we will ask for an opt-out possibility as an alternative or otherwise follow the regular consent procedure. With opt-out, parents are given an opportunity to object to their child's data being used for scientific research. The regular consent procedure requires the consent of the infant's parents before participation in the study. Each site will be responsible for obtaining ethical approval if required by their institution, in collaboration with the study coordinator. We will prioritize centers where a waiver of consent has been granted over centers where an opt-out or regular consent is required to reduce consent-related bias, as described previously.

# 10. ADMINISTRATIVE ASPECTS, MONITORING AND PUBLICATION

## 10.1 Handling and storage of data and documents

Data will be collected, stored, and processed in accordance with the ICH E6 Good Clinical Practise (GCP)- Guidelines. Electronic records will be held on a secure network requiring user ID and password access. Neonates enrolled in the INSPIRE study will be automatically assigned to a study number to encode all data directly after they are included in the study. The pseudonymization key will be safeguarded and kept by the local investigator of each participating center. Regulations between the participating centers and the initiating center about the intended use, confidentiality, security and sharing of the data, and potential financial costs will be arranged in a data sharing agreement (DSA).

Study data will be entered into Castor, a certified electronic database for collection and analysis. Castor complies with all applicable laws and regulations, including ICH E6 Good Clinical Practice (GCP) and by using Castor, researchers are enabled to comply with these laws and regulations (www.castoredc.com). Coded data will be stored in a Leiden University Medical Center ProMISe Datasafe. ProMISe meets the requirements for data safety and privacy set by international law. The ProMISe system facilitates the availability, integrity, and confidentiality of study data, according to the security conditions demanded by GCP. All data is coded and stored on Datasafes, that are exclusively accessible for the involved researchers. The ProMISe Datasafe automatically makes back-ups twice a day. Data will be stored for the length of the study and 15 years afterwards.

## 10.2 Monitoring and Quality Assurance

Data collection procedures will be designed in such a manner as to minimize data entry errors, and we will perform a pilot study to assess the quality of the database. We will monitor the incoming data during the data collection period for inconsistencies, data entry errors, and missing data. Data acquired by this study will be available for inspection by the respective institutional review boards of the participating centers, representatives of national and local health authorities, if applicable, upon request. Given the neglectable risk associated with this observational study, no data safety monitoring board will be installed.

## 10.3 Amendments

Not applicable since amendments to the protocol of non-WMO studies do not have to be submitted to the METC.

## 10.4 End of study report

Not applicable.

## 10.5 Public disclosure and publication policy

The study will be registered on the website of the Dutch National Competent Authority, the ‘Centrale Commissie Mensgebonden Onderzoek’ (CCMO) and the public ClinicalTrials.gov study registry. The results from the International Neonatal Point Prevalence Study will be analysed and published as soon as possible in open access peer-reviewed international scientific journals and presented at scientific meetings unless the study was terminated prematurely and did not yield sufficient data for a publication. The responsibility for presentations and/or publications belongs to the investigators. No restriction regarding the public disclosure and publication of the research data have been or will be made by the funding agencies. All papers written as a part of this study will list the funding agencies. The final publication of the study results will be written by the principal investigators and the co-investigators. National coordinators will be named as co-authors on any publications resulting from this study, local coordinating investigators will be named under the INSPIRE-group authorship (max. 2 investigators per participating center). A draft manuscript will be submitted for review to all co-authors. Results will also be published in a PhD-thesis.

## 11. REFERENCES

1. Keir AK, Stanworth SJ. Neonatal Plasma Transfusion: An Evidence-Based Review. Transfus Med Rev. 2016;30(4):174-82.
2. Franz AR, Engel C, Bassler D, Rüdiger M, Thome UH, Maier RF, et al. Effects of Liberal vs Restrictive Transfusion Thresholds on Survival and Neurocognitive Outcomes in Extremely Low-Birth-Weight Infants: The ETTNO Randomized Clinical Trial. Jama. 2020;324(6):560-70.
3. Kirpalani H, Bell EF, Hintz SR, Tan S, Schmidt B, Chaudhary AS, et al. Higher or Lower Hemoglobin Transfusion Thresholds for Preterm Infants. N Engl J Med. 2020;383(27):2639-51.
4. Bell EF, Strauss RG, Widness JA, Mahoney LT, Mock DM, Seward VJ, et al. Randomized trial of liberal versus restrictive guidelines for red blood cell transfusion in preterm infants. Pediatrics. 2005;115(6):1685-91.
5. Whyte RK, Kirpalani H, Asztalos EV, Andersen C, Blajchman M, Heddle N, et al. Neurodevelopmental outcome of extremely low birth weight infants randomly assigned to restrictive or liberal hemoglobin thresholds for blood transfusion. Pediatrics. 2009;123(1):207-13.
6. Andrew M, Vegh P, Caco C, Kirpalani H, Jefferies A, Ohlsson A, et al. A randomized, controlled trial of platelet transfusions in thrombocytopenic premature infants. J Pediatr. 1993;123(2):285-91.
7. Curley A, Stanworth SJ, Willoughby K, Fustolo-Gunnink SF, Venkatesh V, Hudson C, et al. Randomized Trial of Platelet-Transfusion Thresholds in Neonates. N Engl J Med. 2019;380(3):242-51.
8. Elbourne D. A randomized trial comparing the effect of prophylactic intravenous fresh frozen plasma, gelatin or glucose on early mortality and morbidity in preterm babies. Eur J Pediatr. 1996;155(7):580-8.
9. Scrivens A, Reibel N-J, Heeger LE, Stanworth S, Lopriore E, New H, et al. Survey on neonatal transfusion practices in 18 European countries. Manuscript in preparation. 2021.
10. Stainsby D, Jones H, Wells AW, Gibson B, Cohen H. Adverse outcomes of blood transfusion in children: analysis of UK reports to the serious hazards of transfusion scheme 1996-2005. Br J Haematol. 2008;141(1):73-9.
11. Australian National Blood Authority. Patient Blood Management Guidelines: Module 6 - Neonatal and Paediatrics. Canberra, Australia: NBA; 2016.
12. New HV, Berryman J, Bolton-Maggs PH, Cantwell C, Chalmers EA, Davies T, et al. Guidelines on transfusion for fetuses, neonates and older children. Br J Haematol. 2016;175(5):784-828.
13. Cremer M, Sola-Visner M, Roll S, Josephson CD, Yilmaz Z, Bührer C, et al. Platelet transfusions in neonates: practices in the United States vary significantly from those in Austria, Germany, and Switzerland. Transfusion. 2011;51(12):2634-41.
14. Bruun MT, Yazer MH, Spinella PC, Titlestad K, Lozano M, Delaney M, et al. Vox Sanguinis International Forum on paediatric indications for blood component transfusion: Summary. Vox Sang. 2019;114(5):523-30.
15. Guillén U, Cummings JJ, Bell EF, Hosono S, Frantz AR, Maier RF, et al. International survey of transfusion practices for extremely premature infants. Semin Perinatol. 2012;36(4):244-7.
16. Patel RM, Hendrickson JE, Nellis ME, Birch R, Goel R, Karam O, et al. Variation in Neonatal Transfusion Practice. J Pediatr. 2021.
17. Cure P, Bembea M, Chou S, Doctor A, Eder A, Hendrickson J, et al. 2016 proceedings of the National Heart, Lung, and Blood Institute's scientific priorities in pediatric transfusion medicine. Transfusion. 2017;57(6):1568-81.
18. Weiss SL, Fitzgerald JC, Faustino EV, Festa MS, Fink EL, Jouvet P, et al. Understanding the global epidemiology of pediatric critical illness: the power, pitfalls, and practicalities of point prevalence studies. Pediatr Crit Care Med. 2014;15(7):660-6.

19. Fustolo-Gunnink SF, Roehr CC, Lieberman L, Christensen RD, Van Der Bom JG, Dame C, et al. Platelet and red cell transfusions for neonates: lifesavers or Trojan horses? Expert Rev Hematol. 2019;12(10):797-800.
20. American Academy of Paediatrics. Levels of Neonatal Care. Pediatrics. 2012;130(3):587-97.
21. Chawanpaiboon S, Vogel JP, Moller AB, Lumbiganon P, Petzold M, Hogan D, et al. Global, regional, and national estimates of levels of preterm birth in 2014: a systematic review and modelling analysis. Lancet Glob Health. 2019;7(1):e37-e46.
22. Motta M, Del Vecchio A, Perrone B, Ghirardello S, Radicioni M. Fresh frozen plasma use in the NICU: a prospective, observational, multicentred study. Arch Dis Child Fetal Neonatal Ed. 2014;99(4):F303-8.
23. Altuntas N, Yenicesu I, Beken S, Kulali F, Burcu Belen F, Hirfanoglu IM, et al. Clinical use of fresh-frozen plasma in neonatal intensive care unit. Transfus Apher Sci. 2012;47(1):91-4.
24. Houben NAM, Heeger LE, Stanworth SJ, New HV, van der Bom JG, Fustolo-Gunnink S, et al. Changes in the Use of Fresh-Frozen Plasma Transfusions in Preterm Neonates: A Single Center Experience. J Clin Med. 2020;9(11).
25. Wensing M, Grol R. Implementatie: Effectieve verbetering van de patiëntenzorg: Bohn Stafleu van Loghum; 2017.
26. Weiss EM, Olszewski AE, Guttmann KF, Magnus BE, Li S, Shah AR, et al. Parental Factors Associated With the Decision to Participate in a Neonatal Clinical Trial. JAMA Netw Open. 2021;4(1):e2032106.
27. Vidiella-Martin J, Been JV, Van Doorslaer E, García-Gómez P, Van Ourti T. Association Between Income and Perinatal Mortality in the Netherlands Across Gestational Age. JAMA Network Open. 2021;4(11):e2132124-e.
28. Michel M, Alberti C, Carel JC, Chevreul K. Social inequalities in access to care at birth and neonatal mortality: an observational study. Arch Dis Child Fetal Neonatal Ed. 2021.
29. Stephens BE, Bann CM, Poole WK, Vohr BR. Neurodevelopmental impairment: Predictors of its impact on the families of extremely low birth weight infants at 18 months. Infant Mental Health Journal. 2008;29(6):570-87.
30. Simeoni S, Frova L, De Curtis M. Inequalities in infant mortality in Italy. Italian Journal of Pediatrics. 2019;45(1):11.
31. Damsted Rasmussen T, Villadsen SF, Kragh Andersen P, Smith Jervelund S, Nybo Andersen A-M. Social and ethnic disparities in stillbirth and infant death in Denmark, 2005–2016. Scientific Reports. 2021;11(1):8001.
32. Ravelli AC, Tromp M, Eskes M, Droog JC, van der Post JA, Jager KJ, et al. Ethnic differences in stillbirth and early neonatal mortality in The Netherlands. J Epidemiol Community Health. 2011;65(8):696-701.
33. Wallace ME, Mendola P, Kim SS, Epps N, Chen Z, Smarr M, et al. Racial/ethnic differences in preterm perinatal outcomes. Am J Obstet Gynecol. 2017;216(3):306.e1-.e12.
34. Shastry A, Bajuk B, Abdel-Latif ME. Are we enrolling representative cohorts of premature infants in our clinical trials? Journal of Perinatology. 2022;42(1):86-90.
35. Rich W, Finer NN, Gantz MG, Newman NS, Hensman AM, Hale EC, et al. Enrollment of extremely low birth weight infants in a clinical research study may not be representative. Pediatrics. 2012;129(3):480-4.
36. Patel RM, Hendrickson JE, Nellis ME, Birch R, Goel R, Karam O, et al. Variation in Neonatal Transfusion Practice. J Pediatr. 2021;235:92-9.e4.
37. Heeger LE, Counsilman CE, Bekker V, Bergman KA, Zwaginga JJ, Te Pas AB, et al. Restrictive guideline for red blood cell transfusions in preterm neonates: effect of a protocol change. Vox Sang. 2019;114(1):57-62.
38. Sparger KA, Assmann SF, Granger S, Winston A, Christensen RD, Widness JA, et al. Platelet Transfusion Practices Among Very-Low-Birth-Weight Infants. JAMA Pediatr. 2016;170(7):687-94.

39. Hodson J, Garros C, Jensen J, Duff JP, Garcia Guerra G, Joffe AR. Parental opinions regarding consent for observational research of no or minimal risk in the pediatric intensive care unit. Journal of Intensive Care. 2019;7(1):60.
40. Menon K, Ward RE, Gaboury I, Thomas M, Joffe A, Burns K, et al. Factors affecting consent in pediatric critical care research. Intensive Care Medicine. 2012;38(1):153-9.
41. Menon K, Ward R, Group ftCCCT. A study of consent for participation in a non-therapeutic study in the pediatric intensive care population. Journal of Medical Ethics. 2014;40(2):123-6.
42. Rich WD, Katheria AC. Waiver of Consent in a Trial Intervention Occurring at Birth-How Do Parents Feel? Front Pediatr. 2017;5:56.

**AMENDMENT PROTOCOL**

August 2nd, 2022

As described in Section 6.3 of the protocol (page 20), we have established a parental advisory board for the study, in collaboration with the European Foundation for the Care of the Newborn Infants (EFCNI). The board was invited to provide input for the study. Their feedback indicated that, as parents, they are very concerned about iatrogenic blood loss as a result of blood testing in extremely premature infants. In response to their feedback, we included an additional section on blood testing in the CRF. In this section, we collect the number of blood tests determined in neonates born with a gestational age below 28 weeks. This data is only collect during the first 28 postnatal days or part thereof, depending on the length of hospitalisation/study period.

# STATISTICAL ANALYSIS PLAN

*Platelet Transfusion Paper*

Version 1
January 29th, 2023

**Principal investigators:**
Prof. Dr. E. Lopriore
Prof. Dr. J.G. van der Bom
Prof. Dr. C.J. Fijnvandraat
Dr. S.F. Fustolo-Gunnink
Prof. Dr. Christof Dame
Dr. Charles Roehr
Dr. Emöke Deschmann
Prof. Dr. Simon Stanworth
Dr. Helen New

**Biostatistician:**
Prof. dr. Le Cessie

**Data-manager:**
Dr. Camila Caram Deelder

**Coordinating investigator:**
N.A.M. Houben, PhD student

**ISRCTN registry:**
Registration number ISRCTN17267090

## TABLE OF CONTENTS


**1. STUDY SUMMARY** .... 3

**2. STUDY OUCOME MEASURES** .... 4

*2.1 Main study outcome measures* .... 4

*2.2 Other study outcome measures* .... 4

**3. DATA CLEANING** .... 5

*3.1 Data cleaning during study follow-up* .... 5

*3.2 Data cleaning after completion study follow-up* .... 5

**4. DATA ANALYSES** .... 6

*4.1 General remarks* .... 6

*4.2 Missing data* .... 6

*4.3 Data transformations* .... 6

*4.4 Patient and center characteristics* .... 8

**5. ANALYSIS OF MAIN STUDY OUTCOME MEASURES** .... 9

*5.1 Platelet transfusion day prevalence rates* .... 9

*5.2 Case-mix adjusted platelet transfusion day prevalence rates* .... 9

*5.3 Cumulative incidence of receiving at least one platelet transfusion* .... 11

**6. ANALYSIS FOR OTHER STUDY OUTCOME MEASURES** .... 12

*6.1 Primary indications for platelet transfusion* .... 12

*6.2 Transfusion volume, duration and infusion rate* .... 12

*6.3 Platelet count prior to transfusion* .... 12

*6.4 Platelet transfusion increment* .... 12

*6.5 Transfusion related adverse effects after platelet transfusion* .... 13

**7. GENERAL REMARKS** .... 14

**8. STATEMENT** .... 15

**9. REFERENCES** .... 16

## 1. STUDY SUMMARY

**Study rationale:** Premature infants are a highly transfused group, though robust evidence supporting neonatal transfusion practice is scarce. The PlaNeT-2/MATISSE platelet transfusion trial found that a 25 $x10^9/L$ platelet count threshold was superior to a 50 $x10^9/L$ threshold in preterm infants, with higher mortality and major bleeding rates in the latter liberal threshold group. (1) There are no neonatal transfusion guidelines that have been implemented by Europe as a whole, resulting in significant variation in transfusion practice within Europe. Detailed contemporary data on neonatal transfusion practices in Europe, including the use of platelet transfusions, are lacking.

**Study population:** preterm infants born below 32 weeks gestation admitted to a tertiary level Neonatal Intensive Care Unit (NICU)

**Study design:** prospective, international, multicenter, observational study

**Study data collection:** data collection took place from September 2022 to August 2023. All participating centers collected data during a fixed six-week study period. Local sites documented transfusion use in all infants in their NICU during these weeks, including infants already admitted at the start of the study period or newly admitted during the study period. Consequently, not all infants were followed from birth, and the duration of study follow-up varied, with a maximum follow-up of 42 days per included patient.

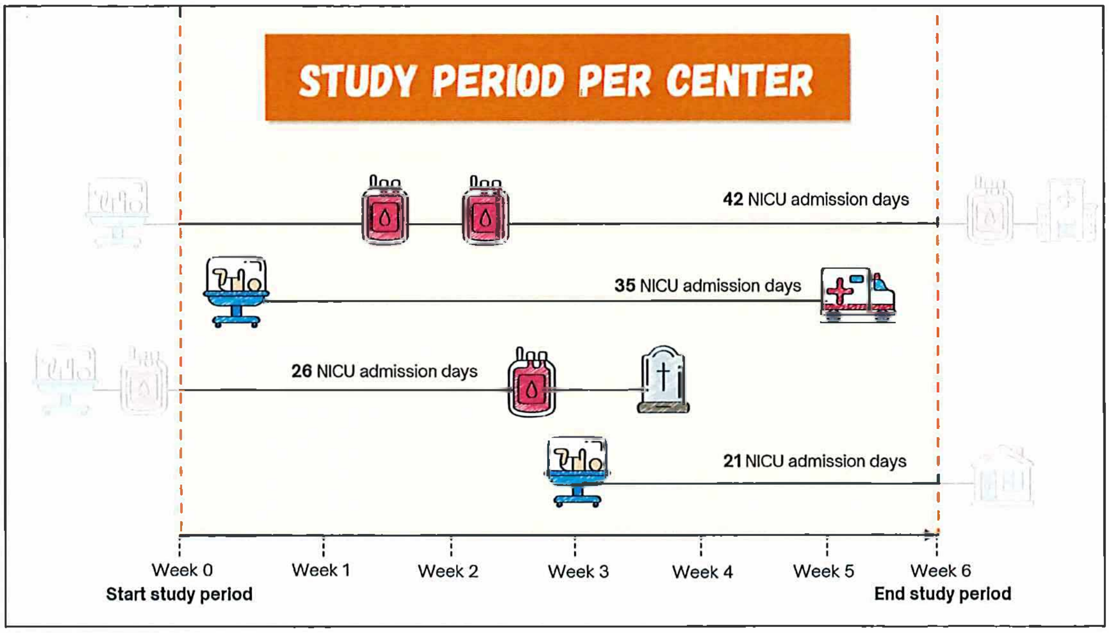


*Infographic was made using images of Juicy_Fish from FlatIcon.*

## 2. STUDY OUCOME MEASURES

*We aim to publish separate papers describing neonatal RBC, platelet and plasma transfusions.*
***Therefore, this SAP only focuses on the use of platelet transfusions.***

### 2.1 Main study outcome measures

1. Platelet transfusion day prevalence rate
2. Case-mix adjusted platelet transfusion day rate
3. Cumulative incidence of receiving at least one platelet transfusion during first 28 days of life

### 2.2 Other study outcome measures

4. Primary indications for platelet transfusion
5. Volume, duration and infusion rate of platelet transfusion
6. Platelet count prior to platelet transfusion
7. Platelet transfusion increment, stratified for transfusion volume
8. Transfusion related adverse effects

## 3. DATA CLEANING

### 3.1 Data cleaning during study follow-up

The site specific patient data was checked and cleaned after the completion of the study period in that participating center. This data quality check was used to detect outliers and impossible combinations of data entry (e.g. non-chronological dates, transfusions after end of study follow-up , etc.). Additionally, we checked for missing data in the baseline characteristics and outcome data. In case of missing data or ambiguities, the local investigator was contacted and incorrect values were directly corrected in the data management system Castor.

### 3.2 Data cleaning after completion study follow-up

A final data quality check will be performed once all the centers completed the study period using the same data quality check code. Possible ambiguities that were overlooked during the center specific check will be corrected, where any modifications made will be recorded in the data cleaning code.

# 4. DATA ANALYSES

## 4.1 General remarks

All statistical analyses will be performed using R statistical software (Version 4.1.17, R Core Team, 2021) or STATA statistical Software (Version 16.1, Texas, USA). Continuous data will be presented as mean (standard deviation (SD)) or as median (interquartile range (IQR)), where appropriate.

## 4.2 Missing data

Missing values were limited in the eCRF since the majority of the collected variables were marked as mandatory to fill in. Therefore, we expect very few missing values in the dataset.

We decided in advance how to handle missing data for the following variables:

| Variables | Commentary |
|---|---|
| **Platelet count prior to transfusion** | Only recorded if platelet count was available in the 24 hours before transfusion. If left blank, the value is recorded as 'not available'. |
| **Platelet count after transfusion** | Only recorded if platelet count was available in the 24 hours after transfusion. If left blank, the value is recorded as 'not available'. |
| **Congenital anomalies** | If not recorded, we assume that congenital anomalies were unlikely and the value is recorded as '0'. |
| **Bleeding disorders** | If not recorded, we assume bleeding disorders were not present and the value is recorded as '0'. |
| **Major bleeding** | If not recorded we assume no major bleeding occurred and the value is recorded as '0'. |
| **NEC** | If not recorded, we assume no NEC occurred and the value is recorded as '0'. |
| **Sepsis** | If recorded as unknown, we assume no sepsis occurred and the value is recorded as '0'. |
| **Mechanical ventilation** | If recorded as unknown, we assume no mechanical ventilation occurred and the value is recorded as '0'. |
| **Surgical procedures** | If recorded as unknown, we assume no surgical procedures occurred and the value is recorded as '0'. |

For all other variables, the total number of missing values will be reported, with the number of infants who had one or more missing values for the variable.

## 4.3 Data transformations

For the clinical events, investigators only collected the start date of clinical events (except mechanical ventilation) so no information is available on the duration of the event or the

time to clinical remission. Therefore, we made assumptions about the duration of clinical events, which allows us to use these variables for the case-mix adjusted prevalence rates.

The variables for clinical events were defined as followed:

| Clinical events | Definitions |
|---|---|
| **Major bleeding** | The duration of a major bleeding episode is defined as 7 days from the day of detection of the major bleeding. For each individual admission day, one of the values will be assigned:<br>– **0:** No major bleeding<br>– **1:** Current bleeding episode<br>– **2.** Status post bleeding |
| **NEC** | The duration of a NEC episode is defined as 7 days from the day of the first symptoms. For each individual admission day, one of the values will be assigned:<br>– **0:** No NEC<br>– **1:** Current NEC episode<br>– **2.** Status post NEC |
| **Sepsis** | The duration of a sepsis episode is defined as 7 days from the day of the positive blood culture. For each individual admission day, one of the values will be assigned:<br>– **0:** No sepsis<br>– **1:** Current sepsis episode<br>– **2:** Status post sepsis |
| **Mechanical ventilation** | The duration of a mechanical ventilation episode is defined as the period from the postnatal day of start mechanical ventilation until the postnatal day of stop mechanical ventilation. For each individual admission day, one of the values will be assigned:<br>– **0:** No mechanical ventilation<br>– **1:** Current mechanical ventilation episode<br>– **2:** Status post mechanical ventilation |
| **Surgical procedures** | The duration of a surgical procedure episode is defined as 7 days from the day of the surgical procedure. For each individual admission day, one of the values will be assigned:<br>– **0:** No surgical procedure<br>– **1:** Current surgy episode<br>– **2:** Status post surgery |

For these assigned values, it is not possible for infants to go back to a status with a lower value, example given from "1" to "0".

> ***Example.*** *Patient A was followed in the study from postnatal day 1 (equal to day of birth) to postnatal day 28, during which the patient developed a culture proven sepsis episode on day 4. In the study database, the patient was represented by 28 rows each representing one admission day in study follow-up. For this sepsis episode, day 4 and the following six days until day 10 were recorded as "1". Prior to sepsis, day 1 to 3 were recorded as "0" (no sepsis), day 11 to 28 as "2" (status post sepsis).*

### 4.4 Patient and center characteristics

The following descriptive data on patient and center characteristics will be presented.

Patient characteristics (for total and per country)

- Number of patient included
- Total number of admission days in study follow-up
- Duration of study follow-up per patient, in days
- Postnatal age at start follow-up in the study, in days
- Sex, % female
- Gestational age at birth, in days
- Birth weight, in grams
- Multifetal pregnancy, % singleton
- Patients with congenital anomalies, %
- Patients with intrauterine growth restriction, %
- Patients with at least one major bleeding during study follow-up, %
- Patients with at least one NEC episode during study follow-up, %
- Patients with at least one sepsis episode during study follow-up, %
- Patients with at least one day of mechanical ventilation during study follow-up, %
- Patients with at least one surgical procedure during study follow-up, %

Participating centers (for total and per country)

- Number of academic centers, %
- Number of centers that perform NEC surgery, %
- Number of larger centers (caring >100 preterm infants with a GA below 32 weeks yearly on average), %

## 5. ANALYSIS OF STUDY OUTCOME MEASURES DESCRIBING PLATELET USE

### 5.1 Platelet transfusion day prevalence rates

The prevalence rate is the rate of events (new and recurring events) over a specified time period. The platelet transfusion day prevalence rate in a certain time period is defined as the number of platelet transfusion days per 100 NICU admission days in this period. A platelet transfusion day is defined as any day after birth on which the infant received at least one platelet transfusion.

$$\textbf{\textit{Prevalence rate}} = \frac{\textit{Number of RBC transfusions days during study period}}{\textit{Number of admission days in follow up during study period}} \times 100\ \textit{days}$$

The platelet transfusion day prevalence rate is calculated in each center and pooled per country. We expect a large between country variability, therefore all prevalence rates are pooled using random effects Poisson models described by Stijnen et al. (2010). This model uses the exact within-study likelihood instead of the approximate normal within-study likelihood which is known to perform better with low event rates and small sample sizes. Additionally, this method does not require the use of an continuity correction in case centers have a prevalence rate of 0 platelet transfusion days. (2) Pooling is done using the function "metarate" of the R-package Meta.

Subgroup rates and overall rate will be presented with 95% confidence intervals in a forest plot. Prevalence rates will only presented on country level.

### 5.2 Case-mix adjusted platelet transfusion day prevalence rates

As variation between countries in platelet prevalence rates could be explained by differences in population characteristics and disease severity, we will calculate case-mix adjusted platelet transfusion day prevalence rates adjusted for several patient demographics and clinical variables to allow fair comparison between prevalence rates.

Case-mix adjusted platelet transfusion day prevalence rates will be calculated as followed:

1. A logistic regression model is fitted on all data, with case-mix variables and clinical variables as (possible time varying) predictors and platelet transfusion as response variable.
2. The model is used to estimate the expected platelet transfusion day prevalence rate per country
3. Observed / expected prevalence rate ratio per country are calculated
4. Rate ratio are multiplied with overall unadjusted platelet prevalence rate

5.2.1. Average expected platelet transfusion day prevalence rate per country

A logistic regression model is used to estimate the probability that a NICU admission day is a platelet transfusion day based on several covariables, for each individual admission day in study follow-up. To account for the repeated measures within an infant, the model is fitted using generalized estimation equations (GEE).
Probabilities are calculated individually for each patient for each NICU admission day in study follow-up and summarized per country to calculate the average predicted platelet transfusion day prevalence rate.

$$\boldsymbol{Average\ expected\ RBC\ transfusion\ day\ prevalence\ rate\ per\ country} = \frac{Sum\ of\ predicted\ probability\ per\ admission\ day\ over\ admission\ days\ over\ infants\ per\ country}{Number\ of\ admission\ days\ per\ country}$$

The following variables are included in the regression analyses. The clinical variables could vary over time and are included under the assumption that experiencing these events leads to an increased risk of transfusion and not vice versa.

- **Baseline variables**: sex, gestational age at birth, birth weight, congenital anomalies, intrauterine growth restriction (birth weight below $10^{th}$ percentile) (3)
- **Clinical variables:** major bleeding, NEC, sepsis, mechanical ventilation, surgical procedure, postnatal day

5.2.2. Observed / expected prevalence rate ratio per country

The prevalence rate ratio per country is defined as the country's observed platelet transfusion day prevalence (as calculated in section 5.1) compared to the country's average expected platelet transfusion day prevalence rate.

$$\textbf{Prevalence rate ratio per country} = \frac{\textit{Observed platelet day prevalence rate per country}}{\textit{Average expected platelet day prevalence rate per country}}$$

Observed / expected prevalence rate ratios will be presented in an observed/expected funnel plot.

5.2.3 Case-mix adjusted prevalence rates

The standardized platelet transfusion day prevalence rates are defined as the prevalence rate ratio for a country multiplied with the overall unadjusted platelet prevalence rate.

$$\textbf{Case} - \textbf{mix adjusted prevalence rate per country} = \textit{Prevalence rate ratio per country} * \textit{overall unadjusted platelet prevalence rate}$$

**5.3 Cumulative incidence of receiving at least one platelet transfusion**

The cumulative incidence (with 95% confidence interval) of receiving at least one platelet transfusion during the first 28 days of life will be calculated where death and discharge are considered as competing events. Infants that were already present at the NICU at the start of the study period are excluded, because the first platelet transfusion for these infants could have happened before the study period. Event time for infants who were transferred or discharged during the first 28 days of life were censored. The cumulative incidence was calculated in R with the package "cmprsk" using function "cuminc".

# 6. ANALYSIS FOR OTHER STUDY OUTCOME MEASURES

## 6.1 Primary indications for platelet transfusion

We will present the primary indications for platelet transfusion. The indications reported under 'other indications' will be categorized where possible at the discretion of the primary investigators and will be reported.

## 6.2 Transfusion volume, duration and infusion rate

Transfusion infusion rates will be calculated by dividing the volume (mL/kg) by the transfusion duration (in hours). Transfusion infusion rates will be presented in two bubble plots, separately for platelet transfusions given based on platelet count threshold and platelets transfusion given for active bleeding, prevention of major bleeding, lumbar puncture, surgical procedure and any other primary indications.

## 6.3 Platelet count prior to transfusion

We collected the last platelet count measured prior to platelet transfusion, if available within maximum 24 hours before transfusion.

The available platelet count values prior to transfusion will be presented in two box plots, separately for the platelet transfusions given based on platelet count threshold and platelets transfusion given for active bleeding, prevention of major bleeding, lumbar puncture, surgical procedure and any other primary indications.

We will present the proportion of platelet counts prior to transfusions given based on threshold that is below the restrictive threshold ($<25 \times 10^9$/L), between the restrictive and below the liberal threshold ($25 \times 10^9$/L to $<50 \times 10^9$/L), and above the liberal threshold ($\geq 50 \times 10^9$/L) as compared in the PlaNeT2-Matisse trial. (1)

## 6.4 Platelet transfusion increment

We also collected the post-transfusion platelet count at the most recent measurement after the platelet transfusion, if available within maximum 24 hours after transfusion. All platelet transfusions given based on platelet count threshold in which both pre-transfusion and post-transfusion platelet count are available will be used for the following plots. Transfusion increment will be calculated by subtracting post-transfusion platelet count from pre-transfusion platelet count.

Transfusion increments will be plotted separately for the different transfusion volume categories as well as for all transfusions. Transfusion increment of a transfusion volume group will not be plotted if 5 or less platelet transfusions given at this volume.

**6.5 Transfusion related adverse effects following platelet transfusion**

In the absence of clear definitions of transfusion-associated side effects in preterm infants, local investigators were asked to register any perceived transfusion-associated adverse effects if they consider the adverse event to be potentially associated with the preceding transfusion.

We will report the percentage of platelet transfusions for which an adverse events was registered and the mean/median time between occurrence of the adverse effect and the platelet transfusion, in days. The observed adverse effects will be categorized where possible at the discretion of the primary investigators and will be reported in text.

## 7. GENERAL REMARKS

### Reporting guidelines

The study results will be presented according to the recommendations of the STROBE guidelines.

### Publication

The statistical analysis plan will be published in the ISRCTN registry.

## 8. STATEMENT

We hereby declare that this statistical analysis plan has been drafted prior to analysis of the INSPIRE platelet data for the manuscript, and that this plan will be followed during the analysis phase. If any deviations from the plan are made, they will be incorporated and explained in an amendment.

**Date:**

**Version:**

**Principal investigator:**
Prof. dr. E. Lopriore

**Biostatistician :**
Prof. dr. Le Cessie

**Coordinating investigator:**
Nina Houben

## 9. REFERENCES

1. Curley A, Stanworth SJ, Willoughby K, Fustolo-Gunnink SF, Venkatesh V, Hudson C, et al. Randomized Trial of Platelet-Transfusion Thresholds in Neonates. N Engl J Med. 2019;380(3):242-51.

2. Stijnen T, Hamza TH, Ozdemir P. Random effects meta-analysis of event outcome in the framework of the generalized linear mixed model with applications in sparse data. Stat Med. 2010;29(29):3046-67.

3. Hoftiezer L, Hof MHP, Dijs-Elsinga J, Hogeveen M, Hukkelhoven C, van Lingen RA. From population reference to national standard: new and improved birthweight charts. Am J Obstet Gynecol. 2019;220(4):383.e1-.e17.

## Supplement 3. INSPIRE Study Group (non-author collaborators)

| First Name | Last name | Affiliation | Location | Role |
|---|---|---|---|---|
| Miguel | Alsina-Casanova | Clínic Barcelona Hospital Universitari | Barcelona, Spain | Local investigator |
| Ola | Andersson | Lund University and Skane University Hospital | Lund, Sweden | Local investigator |
| Rosa Patricia | Arias-Llorente | Central University Hospital of Asturias | Oviedo, Spain | Local investigator |
| Adeline | Berenger | CHU Limoges | Limoges, France | Local investigator |
| Edyta | Bielska | Department of Neonatology and Neonatal Intensive Care Medical University of Warsaw | Warsaw, Poland | Local investigator |
| Marioara | Boia | Spitalul Clinic de Urgenta Pentru Coppii Louis Turcanu | Timisoara, Romania | Local investigator |
| André | Birkenmaier | University of Zurich and Children's Hospital St. Gallen | St. Gallen, Switzerland | Local investigator |
| Jakub | Biros | Faculty Hospital Nove Zamky | Nove Zamky, Slovakia | Local investigator |
| Anne Laure | Blanquart | CHU Limoges | Limoges, France | Local investigator |
| Tiziana | Boggini | Fondazione IRCCS Policlinico San Matteo | Pavia, Italy | Local investigator |
| Pascal | Boileau | CHI Poissy - Saint-Germain-en-Laye | Poissy, France | Local investigator |
| Renata | Bokiniec | Department of Neonatology and Neonatal Intensive Care Medical University of Warsaw | Warsaw, Poland | Local investigator |
| Ilia | Bresesti | Filippo Del Ponte Hospital, University of Insubria | Varese, Italy | Local investigator |
| Katherine | Broad | St Michael's Hospital, University Hospitals Bristol and Weston | Bristol, United Kingdom | Local investigator |
| Giacomo | Cavallaro | Neonatal Intensive Care Unit, Fondazione IRCCS Ca' Granda Ospedale Maggiore Policlinico | Milano, Italy | Local investigator |
| Jennifer | Chauvel | CH de Saint Brieuc | Saint-Brieuc, France | Local investigator |
| Borbála | Cseszneki | Semmelweis University | Budapest, Hungary | Local investigator |
| Carlo | Dani | Azienda Ospedaliero Universitaria Careggi | Florence, Italy | Local investigator |
| Klaudia | Demová | Faculty Hospital Nove Zamky | Nove Zamky, Slovakia | Local investigator |
| Diana | Dornis | Universitätsklinikum Leipzig | Leizpig, Germany | Local investigator |
| Marie-Pierre | Duban | CH de Saint Brieuc | Saint-Brieuc, France | Local investigator |

| | | | | |
|---|---|---|---|---|
| Karolina | Dziadkowiec-Motyl | Górnośląskie Centrum Zdrowia Dziecka, Medical Faculty Silesian Medical University | Katowice, Poland | Local investigator |
| Nika | Erzen | University Medical Center Ljubljana | Ljubljana, Slovenia | Local investigator |
| Eszter | Fanczal | Semmelweis University | Budapest, Hungary | Local investigator |
| Sara | Fernández-Castiñeira | Central University Hospital of Asturias | Oviedo, Spain | Local investigator |
| Libusa | Galuschka | Klinikum Lüneburg | Lüneburg, Germany | Local investigator |
| Ellen | Gandaputra | Klinikum Lüneburg | Lüneburg, Germany | Local investigator |
| Fermín | García-Muñoz Rodrigo | CHU Insular-Materno Infantil | Las Palmas, Spain | Local investigator |
| Corinna | Gebauer | Universitätsklinikum Leipzig | Leizpig, Germany | Local investigator |
| Hélène | Grimault | CH Bretagne-Atlantique | Vannes, France | Local investigator |
| Kristina | Grund | Department of Neonatology, University Children's Hospital Regensburg (KUNO), Hospital St. Hedwig of the Order of St. John, University of Regensburg | Regensburg, Germany | Local investigator |
| Melanie | Gsöllpointner | Kepler University Hospital | Linz, Austria | Local investigator |
| Silvia | Gualdi | IRCCS AOU S. Orsola | Bologna, Italy | Local investigator |
| Brunetta | Guaragni | Spedali Civili di Brescia | Brescia, Italy | Local investigator |
| Markus | Hahn | University of Zurich and Children's Hospital St. Gallen | St. Gallen, Switzerland | Local investigator |
| Nadja | Haiden | Kepler University Hospital | Linz, Austria | Local investigator |
| Monica | Hasmasanu | University of Medicine and Pharmacy Iuliu Hatieganu | Cluj-Napoca, Romania | Local investigator |
| Daniela | Iacob | Timisoara County Emergency Clinical Hospital | Timisoara, Romania | Local investigator |
| Mihaela | Ivanici | Spitalul Clinic de Urgenta Pentru Coppii Louis Turcanu | Timisoara, Romania | Local investigator |
| Raphaela | Jernej | Medical University Vienna | Vienna, Austria | Local investigator |
| Tomáš | Juren | University Hospital Brno | Brno, Czech Republic | Local investigator |
| Karolina | Karcz | Department of Neonatology Wroclaw Medical University | Wrocław, Poland | Local investigator |
| Lilijana | Kornhauser | Maternity Hospital Ljubljana | Ljubljana, Slovenia | Local investigator |
| Barbara | Królak-Olejnik | Department of Neonatology Wroclaw Medical University | Wrocław, Poland | Local investigator |

| | | | | |
|---|---|---|---|---|
| Lena | Legnevall | Karolinska Institute | Stockholm, Sweden | Local investigator |
| Verena | Lehnerer | Department of Neonatology, University Children's Hospital Regensburg (KUNO), Hospital St. Hedwig of the Order of St. John, University of Regensburg | Regensburg, Germany | Local investigator |
| Emmanuelle | Levine | CHU de Rennes | Rennes, France | Local investigator |
| David | Ley | Lund University and Skane University Hospital | Lund, Sweden | Local investigator |
| María Del Carmen | López Castillo | Hospital Regional Universitario de Málaga | Málaga, Spain | Local investigator |
| Mariella | Magarotto | Azienda Ospedale Università Padova | Padova, Italy | Local investigator |
| Silvia | Martini | IRCCS AOU S. Orsola | Bologna, Italy | Local investigator |
| Iwona | Maruniak-Chudek | Górnośląskie Centrum Zdrowia Dziecka, Medical Faculty Silesian Medical University | Katowice, Poland | Local investigator |
| Rita | Moita | CHU de São João | Porto, Portugal | Local investigator |
| Anjola | Mosuro | Great Western Hospitals | Wiltshire, United Kingdom | Local investigator |
| Agnieszka | Nowicka | Centrum Medyczne Ujastek, Kraków | Kraków, Poland | Local investigator |
| Daniel | O'Reilly | Rotunda Hospital | Dublin, Ireland | Local investigator |
| Manuela | Pantea | Timisoara County Emergency Clinical Hospital | Timisoara, Romania | Local investigator |
| Alejandro | Pérez-Muñuzuri | Hospital Clínico Universitario de Santiago | Santiago de Compostela, Spain | Local investigator |
| Tina | Perme | Maternity Hospital Ljubljana | Ljubljana, Slovenia | Local investigator |
| Laura | Picciau | Spedali Civili di Brescia | Brescia, Italy | Local investigator |
| Sandra | Prins | Emma Children’s Hospital Amsterdam University Medical Center, Department of Neonatology | Amsterdam, The Netherlands | Local investigator |
| Maurizio | Radicioni | Azienda Opsedaliera di Perugia | Perugia, Italy | Local investigator |
| Genny | Raffaeli | Neonatal Intensive Care Unit, Fondazione IRCCS Ca' Granda Ospedale Maggiore Policlinico | Milano, Italy | Local investigator |
| Reyes | Roldan-López | Hospital Regional Universitario de Málaga | Málaga, Spain | Local investigator |
| Jean-Michel | Roué | CHRU Brest | Brest, France | Local investigator |
| Beata | Rzepecka Węglarz | Centrum Medyczne Ujastek, Kraków | Kraków, Poland | Local investigator |

| | | | | |
|---|---|---|---|---|
| Greta | Sibrecht | II Department of Neonatology, Poznan University of Medical Sciences | Poznan, Poland | Local investigator |
| Pauline | Snijder | Erasmus MC | Rotterdam, The Netherlands | Local investigator |
| Mirta | Starčević | University Hospital Centre Zagreb | Zagreb, Croatia | Local investigator |
| Emese | Szántó | Semmelweis University | Budapest, Hungary | Local investigator |
| Liliana | Teixeira | Centro Materno-Infantil do Norte - Unidade Local de Saúde de Santo António | Porto, Portugal | Local investigator |
| Laura | Torrejon | La Fe University Hospital | Valencia, Spain | Local investigator |
| Lourdes | Urquía Martí | CHU Insular-Materno Infantil | Las Palmas, Spain | Local investigator |
| Laurien | Vanbuggenhout | UZ Leuven | Leuven, Belgium | Local investigator |
| Lorenzo | Zanetto | Azienda Ospedale Università Padova | Padova, Italy | Local investigator |
